# Computational analysis of cancer cell adhesion in curved vessels affected by wall shear stress for prediction of metastatic spreading

**DOI:** 10.3389/fbioe.2024.1393413

**Published:** 2024-05-27

**Authors:** Nahid Rahmati, Nima Maftoon

**Affiliations:** ^1^ Department of Systems Design Engineering, University of Waterloo, Waterloo, ON, Canada; ^2^ Centre for Bioengineering and Biotechnology, University of Waterloo, Waterloo, ON, Canada

**Keywords:** computational biophysics, metastasis, cancer models, microvessel configuration, cell adhesion

## Abstract

**Introduction:** The dynamics of circulating tumor cells (CTCs) within blood vessels play a pivotal role in predicting metastatic spreading of cancer within the body. However, the limited understanding and method to quantitatively investigate the influence of vascular architecture on CTC dynamics hinders our ability to predict metastatic process effectively. To address this limitation, the present study was conducted to investigate the influence of blood vessel tortuosity on the behaviour of CTCs, focusing specifically on establishing methods and examining the role of shear stress in CTC-vessel wall interactions and its subsequent impact on metastasis.

**Methods:** We computationally simulated CTC behaviour under various shear stress conditions induced by vessel tortuosity. Our computational model, based on the lattice Boltzmann method (LBM) and a coarse-grained spectrin-link membrane model, efficiently simulates blood plasma dynamics and CTC deformability. The model incorporates fluid-structure interactions and receptor-ligand interactions crucial for CTC adhesion using the immersed boundary method (IBM).

**Results:** Our findings reveal that uniform shear stress in straight vessels leads to predictable CTC-vessel interactions, whereas in curved vessels, asymmetrical flow patterns and altered shear stress create distinct adhesion dynamics, potentially influencing CTC extravasation. Quantitative analysis shows a 25% decrease in the wall shear stress in low-shear regions and a 58.5% increase in the high-shear region. We observed high-shear regions in curved vessels to be potential sites for increased CTC adhesion and extravasation, facilitated by elevated endothelial expression of adhesion molecules. This phenomenon correlates with the increased number of adhesion bonds, which rises to approximately 40 in high-shear regions, compared to around 12 for straight vessels and approximately 5–6 in low-shear regions. The findings also indicate an optimal cellular stiffness necessary for successful CTC extravasation in curved vessels.

**Discussion:** By the quantitative assessment of the risk of CTC extravasation as a function of vessel tortuosity, our study offers a novel tool for the prediction of metastasis risk to support the development of personalized therapeutic interventions based on individual vascular characteristics and tumor cell properties.

## 1 Introduction

Cancer metastasis is one of the most challenging and enduring issues in oncology, significantly contributing to cancer-associated death on a global scale (Chaffer and Weinberg, 2011). While treatment methods are frequently effective in controlling primary tumors, the primary factor responsible for cancer-related mortalities lies in the process of metastasis, where cancer spreads to distant organs and tissues. The metastatic cascade represents a complex, multi-stage process in the advancement of cancer disease (Sugarbaker, 1979; Lambert et al., 2017). It begins with the formation of a primary tumor, a site where cancer cells proliferate. At this stage, these cells locally degrade the extracellular matrix, thereby enabling them to infiltrate the neighbouring tissues. Subsequently, these invasive cancer cells have the ability to intravasate into both the bloodstream and lymphatic vessels, ultimately becoming circulating tumor cells (CTCs) (Anvari et al., 2022). This phase presents numerous challenges for CTCs, including exposure to mechanical forces like shear stress and immune surveillance (Wirtz et al., 2011; Anvari et al., 2021). Should CTCs succeed in establishing successful adhesion to the vessel walls, they can then proceed to extravasate into new tissues while adapting to the unique microenvironment of the host tissue (Chambers et al., 2002). This adaptation is a crucial step that ultimately leads to the formation of secondary tumors.

Given that the metastatic cascade stands as the primary driver of cancer-related mortality, our understanding and predictive capabilities of the cascade are of paramount importance to enable therapeutic interventions targeting its various phases. Recent studies (Gallicchio et al., 2022; Hudock et al., 2023) indicate a notable decrease in metastatic cancer incidence rates in the United States, with an average annual percent change (AAPC) decrease of 0.80 per 100,000 individuals from 1988 to 2018 and a projected further decrease of 0.70 per 100,000 individuals until 2040. Notably, metastases to critical organs are forecasted to decline. Additionally, survival rates for metastatic cancer patients are improving, attributed to advancements in therapy. Therefore, this study aims to shed light on a particular aspect of this complexity by investigating how shear stress influenced by vascular architecture impacts the attachment and tendency of CTCs to spread in these vessels, particularly in curved vessels that exhibit twisting and bending. Addressing this enhances our ability to predict metastatic progression and may facilitate the development of personalized therapeutic strategies.

Shear stress, characterized as the tangential mechanical force per unit area exerted by the blood flow on the endothelial cells lining the interior of blood vessels, can have a substantial influence on the fate of CTCs. Numerous studies have been conducted on the impact of shear stress on CTCs, revealing alterations in endothelial cell transport properties that affect CTC survival and proliferation (Tarbell, 2010; Wirtz et al., 2011). Studies have demonstrated that fluid shear stress induces epithelial-mesenchymal transition (EMT) in CTCs, improving their survival under shear flow through the activation of Jun N-terminal kinase (JNK) signalling. Mitchell et al. (2015) observed cell necrosis occurring at shear stress values as low as 6 dynes/cm^2^. In addition, elevated shear stress on CTCs potentially leading to both cell fragmentation and anoikis, contributing to the potential death of CTC in the bloodstream (Headley et al., 2016; Regmi et al., 2017; Wang et al., 2018). Regmi et al. (2017) reported high shear stress inducing apoptosis in cells within the range of 40–60 dynes/cm^2^. The progression of CTCs in the metastasis cascade toward extravasation is intricately linked to their ability to overcome shear forces. Specifically, a critical shear force magnitude per square centimetre in the “intermediate” range, estimated to be between 10 and 20 dyn (Follain et al., 2018), was shown to enable CTCs to adeptly overcome and exploit shear forces and to enhance the likelihood of CTC adhesion to the vascular endothelial cell surface under blood flow (Kang et al., 2016). Chang et al. (2008) demonstrated that a shear stress of 12 dynes/cm^2^ induces CTC adhesion.

This shear-resistant adhesion constitutes an essential factor for cell extravasation, facilitating the successful exit of CTCs from the bloodstream. Consequently, the shear-resistant adhesion of CTCs significantly contributes to their potential for tissue invasion and metastatic dissemination, elucidating a key mechanism in the metastatic cascade. These highlight the intricate impact of mechanical factors, specifically shear stress of blood flow, on various aspects of CTC behaviour.

The configuration and curvature of microvessels can significantly impact the wall shear stress exerted by blood flow. As blood flows through the curved vessel, the curvature induces dynamic alteration in the blood flow pattern, resulting in a distribution of shear stress variations. This dynamic shear stress leads to various mechanical forces exerted on the vessel wall. The elevated shear stress within the inner wall of a curved microvessel has the potential to activate certain signaling pathways in endothelial cells, promoting cell adhesion. Prior studies have shown that this phenomenon induces the unfolding of adhesion proteins like von Willebrand factor, exposing the binding sites to facilitate platelet and leukocyte adhesion (Schneider et al., 2007; Rahmati et al., 2024; Rahmati and Maftoon, 2024). Furthermore, numerous *in vivo* investigations have revealed that the adherence of CTCs is heightened in curved microvessels and complex capillaries such as bifurcated ones in comparison to straight vessels (Liu et al., 2008; Yan et al., 2012; Guo et al., 2014; Zhang et al., 2014). This enhancement is attributed to both the increased shear stress and flow disturbances induced by the curvature.

In addition, there are several numerical studies that highlight the effect of curvature on cell adhesion. Yan et al. (2010; Yan et al., 2012) demonstrated that the vessel curvature significantly influences the wall shear stress affecting the CTC adhesion and significant difference in the number of formed adhesion bonds in comparison to the straight vessels. However, the two-dimensional nature and the simplification of CTCs as rigid disks, overlooking the actual deformability of cells, limit the applicability of their numerical model. Cui et al. (2021) simulated the CTC behavior in curved microvessels, revealing increased bond formation with higher driving forces, indicating the role of centrifugal force. Moreover, their findings revealed that the adhesive interaction between a CTC and the endothelium depend on the cell deformability, with rigid CTCs forming firm adhesions and softer CTCs exhibiting rolling behaviour in the presence of red blood cells (RBCs), potentially enhancing their survival in the bloodstream (Lenarda et al., 2019). Additionally, Xiao et al. (2021) investigated the influence of curvature, cell deformability, and the presence of red blood cells (RBCs) on the motion of a single tumor cell in a low Reynolds number regime. Their work emphasized the dominance of viscous forces and minimal effects of secondary flow. Their findings indicated that higher cell deformability, under low Reynold number, enlarged the contact area, resulting in more formed bonds and prolonged cell arrest while the presence of RBCs increased the drag force, reducing the contact time and causing the adherent tumor cell to quickly move away from the vessel wall.

In this paper, we used a biophysics-based computational model with *a priori* material properties to investigate the CTC adhesion and motion as a result of the wall shear stress variation in curved vessels. Unlike previous studies, our model uniquely incorporates both the deformability of CTCs and the dynamic effects of wall shear stress on endothelial cell ligand exposure, as well as the formation and breakage of bonds, thereby enhancing our understanding of these critical factors. While this study seeks to elucidate the mechanical and structural factors that influence cancer metastasis by investigating the effects of the wall shear stress on the CTC adherence in curved microvessels, it also undertakes a comprehensive analysis of the mechanical and geometric behaviour of CTCs under adhesion to curved vessels, including measurements such as cell deformation by aspect ratio, total adhesion force, temporal and spatial cell velocity, and evaluation of wall shear stress after adhesion. The numerical simulation provided a better understanding of the initiation of extravasation by investigating the underlying adhesion process between CTCs and endothelial cells in curved vessels of various tortuosity indices (TI). The TI is defined as the ratio of the curve length along the vessel’s centerline to the linear distance between the two endpoints. Furthermore, this research contributes to the development of predictive tools for CTC extravasation in vascular structures for evaluating metastasis risk, and disease progression.

## 2 Materials and methods

**FIGURE 1:**
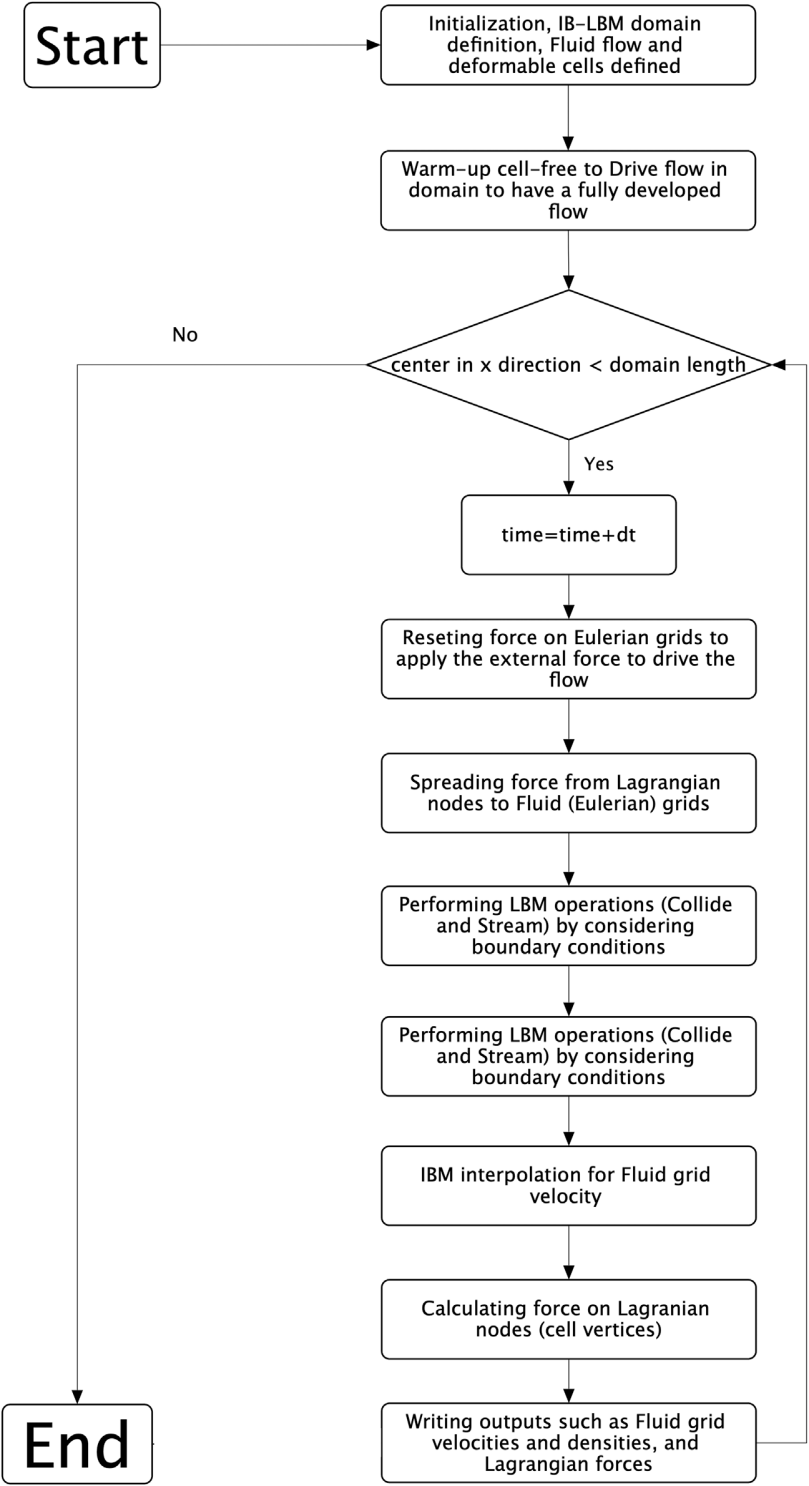
The flowchart illustrating the algorithm employed in the present study.

Within the microcirculatory system, the blood rheological properties are intricately linked to cellular dynamics and the interplay between cells within the bloodstream. To examine the behaviour of single cancerous cells in curved microvessels, we studied deformable CTCs moving within the viscous flow of plasma. The blood plasma was modeled using the lattice Boltzmann method (LBM), implemented in the Palabos open-source code (Version 2.3) (Latt et al., 2020) under the assumption of incompressible Newtonian fluid behavior. CTCs as deformable bodies were modeled using the solid discrete element method (DEM). The deformable cells interacted with the plasma flow employing the immersed boundary method (IBM) (Peskin, 2002), employing the HemoCell open-source code (Version 2.6)(Závodszky et al., 2017; Azizi Tarksalooyeh et al., 2018; Tarksalooyeh et al., 2019; Závodszky et al., 2019), which was augmented in-house be developing a cell-adhesion model. The algorithm used to model the interaction between the fluid and the deformable cells is depicted in the flowchart presented in Figure 1.

### 2.1 Modeling plasma flow using lattice Boltzmann method

The blood plasma flow was simulated through the utilization of the LBM, an alternate method for directly solving the Navier-Stokes Equation. LBM originated from the lattice gas automata (LGA) method for computational fluid dynamics simulations (Chen and Doolen, 1998; Anvari, Osei, and Maftoon, 2021). LBM provides notable advantages compared to traditional computational fluid dynamics (CFD) techniques including finite volume, finite difference, and finite element methods when simulating microscale phenomena, especially in the context of blood flow interactions at the cellular level. Unlike Conventional CFD methods, which depend on the assumption of a continuous fluid and involve discretizing conservation equations, LBM can easily handle complex geometries encountered in microscale simulations by employing a lattice-based framework. In addition, LBM offers advantages in modelling multiphase flows, particularly crucial for blood flow simulations requiring precise representation of cell-level interactions and intricate geometries (Arabghahestani et al., 2019). LBM computes the velocity and density of fluid at every node on the Eulerian grid within the domain of flow. Generally, the basis of the Lattice-Boltzmann equation is attributed to the work of Bhatnagar, Gross, and Krook (BGK) (Bhatnagar et al., 1954), is as follow:

$$f_{i}\left(\vec{r}+{c_{k}\vec{e}}_{i}\delta t,t+\delta t\right)=f_{i}\left(\vec{r},t\right)+\frac{\delta t}{\tau }\left({f_{i}^{eq}\left(\vec{r},t\right)-f}_{i}\left(\vec{r},t\right)\right),$$ (1)

where $f_{i}\left(\vec{r},t\right)$ is the probability of the existence of a particle at position $\vec{r}$ , and time t , ${\vec{e}}_{i}$ is the velocity direction, $c_{k}=\delta x/\delta t$ is the lattice speed, τ is the relaxation time, and $f_{i}^{eq}$ denotes the equilibrium distribution function (Mohamad, 2011). The fluid density ρ and the velocity $\vec{u}$ can be calculated using the zeroth and first moments of the distribution function, as follows:

$$\rho \left(\vec{r},t\right)=\sum_{i}f_{i}\left(\vec{r},t\right),$$ (2)

$$\vec{u}\left(\vec{r},t\right)=\frac{1}{\rho \left(\vec{r},t\right)}\sum_{i}c_{k}{\vec{e}}_{i}f_{i}\left(\vec{r},t\right),$$ (3)

The equilibrium distribution function, denoted as $f_{i}^{eq}$ , is formulated as follows:

$$f_{i}^{eq}\left(\vec{r},t\right)=\omega_{p}\rho \left(1+\frac{{c_{k}\vec{e}}_{i}.\vec{u}}{c_{s}^{2}}+\frac{{\left({c_{k}\vec{e}}_{i}.\vec{u}\right)}^{2}}{2c_{s}^{4}}-\frac{{\vec{u}}^{2}}{2c_{s}^{2}}\right)$$ (4)

Here, $c_{s}$ represents the lattice speed of sound, calculated as of $c_{k}/\sqrt{3}$ , and $\omega_{p}$ denotes the grid-dependent weight values (Mohamad, 2011). Based on Enskog-Chapman analysis (Jehring et al., 1992) of the limit of long wavelengths, for an incompressible flow, the Navier-Stokes equations can be related to LBM equations through the kinematic viscosity, expressed as expressed as $\nu =c_{s}^{2}\left(\tau -\delta t/2\right)$ , and an ideal equation of state of $P=\rho c_{s}^{2}$ . Additional information on the mathematical framework of the LBM is available in other studies (Mohamad, 2011; Závodszky and Paál, 2013).

Furthermore, the plasma and cytoplasm were assumed to be a Newtonian Fluid. In this approach, the quasi-steady nature of cell deformation, with changes occurring gradually over time, was assumed. As a result, the impact of cytoplasm viscosity on cell dynamics was considered negligible, and we applied the same viscosity to both plasma and cytoplasm (Takeishi et al., 2016; Dabagh and Randles, 2019; Dabagh et al., 2020).

### 2.2 Modeling cellular deformations using discrete element method

The modeling of floating deformable bodies such as CTCs within the plasma flow was conducted using the coarse-grained spectrin link membrane method (Závodszky et al., 2017), originally introduced by Fedosov et al. (2010b; Fedosov et al., 2010a). However, there are alternative techniques such as the continuum model, for instance, the finite element method (FEM), which relies on complex constitutive laws for membrane descriptions to capture cell deformation. While DEM offers computational efficiency compared to molecular dynamics techniques (Kotsalos et al., 2019), it is important to note that it can be more expensive than FEM due to the presence of more parameters. Nonetheless, DEM presents several advantages. Notably, it maintains numerical stability and ease of implementation compared to FEM. Moreover, due to the continuity of membrane properties in FEM, the method is limited to modeling at length scales where local property differences in the membrane are insignificant. In contrast, DEM excels in capturing local property differences and heterogeneities, allowing for a more accurate representation of membrane behaviour at various length scales (Silva et al., 2024).

In this methodology, the deformable cell is represented as a solid membrane segmented into triangular elements, where its deformations are modulated by reaction forces acting on membrane nodes. The coarse-grained spectrin-link membrane model integrates four force types, each characterizing specific mechanical behaviours. These forces include $F_{link\;}$ (response to stretching and compression), $F_{bend}$ (reaction to the relative bending of two adjacent triangular elements), $F_{area}$ (the local surface conservation force), and the $F_{volume}$ (the volume conservation force). These forces consist of linear terms with variable slopes for small deformations and fast-diverging nonlinear terms activated during significant deformations, primarily governed by *τ* values to prevent computational instabilities under large deformations. When deformable cells undergo gradual deformation, only the linear term has a noticeable impact, whereas the effect of the nonlinear component is negligible. The mathematical formulation of these four forces are as follows (Závodszky et al., 2017):

$$F_{link\;}=-\frac{k_{l}dL}{p}\left[1+\frac{1}{\tau_{l}^{2}-{dL}^{2}}\right]\;;\;\tau_{l}=3.0,$$ (5)

$$F_{bend\;}=-\frac{k_{b}d\theta }{L_{0}}\left[1+\frac{1}{\tau_{b}^{2}-{d\theta }^{2}}\right]\;;\;\tau_{b}=\frac{\pi }{6},$$ (6)

$$F_{area\;}=-\frac{k_{a}dA}{L_{0}}\left[1+\frac{1}{\tau_{a}^{2}-{dA}^{2}}\right]\;;\;\tau_{a}=0.3,$$ (7)

$$F_{volume\;}=-\frac{k_{v}dV}{L_{0}}\left[1+\frac{1}{\tau_{v}^{2}-{dV}^{2}}\right]\;;\;\tau_{v}=0.01,$$ (8)

Here, the persistence-length of a spectrin filament is denoted as p and equivalent to 7.5 nm (Dao et al., 2003), $L_{0}$ denotes the equilibrium length of the surface element. In the above equations, $dL=\left({L_{i}-L}_{0}\right)/L_{0}$ signifies the edge normal strain, $d\theta =\theta_{i}-\theta_{0}$ , $dA=\left({A_{i}-A}_{0}\right)/A_{0}$ represents the local area strain, and $dV=\left({V_{i}-V}_{0}\right)/V_{0}$ denotes the global volume strain. Additionally, the spring stiffness coefficients ( $k_{l}$ , $k_{b}$ , $k_{a}$ , and $k_{v}$ ) rely on the material characteristics and can be adjusted by the mechanical single-cell experimental results. These constants have been previously determined and calibrated for RBCs and PLTs through optical tweezers experiment (Suresh et al., 2005a; Závodszky et al., 2017).

The mechanical properties of CTCs vary among different types of cancers and their malignancies (Guck et al., 2005; Yu et al., 2011). To simulate the CTC, we have utilized the given constitutive equations, employing the parameter values outlined in Supplementary Table S1. To define the mechanical properties of the cell in our model, we utilized a simulation approach introduced by Závodszky et al. (2017) that involved deforming a singular hexagonal patch of the membrane (see Supplementary Figure S1). The uniaxial stretching of the aforementioned patch based on the spring stiffness coefficients given in Supplementary Table S1 results in a surface Young’s modulus of E_s_ = 264.42 μN/m, and the shear deformation of the patch yields 90 μN/m. The computed mechanical properties for the CTC are approximately nine times greater than those reported for RBCs in Závodszky et al. (2017). In addition, the area expansion of the CTC hexagonal patch results in a compression modulus of K = 247.8 μN/m, which aligns with the reported range of 20–4020 μN/m (Tan et al., 2018). Under the assumption of homogeneous isotropic linear behaviour, which is only valid for minor deformations, the relationship between the elastic constants results in a Poisson’s ratio of 0.337, which is close to the expected value of 1/3 (Omori et al., 2011; Tan et al., 2018).

### 2.3 Model of cellular interaction with plasma using immersed boundary method

To model the interactions between the blood plasma and deformable bodies, we employed the immersed boundary method (IBM) to address the misalignment between the structured Eulerian grid for fluid nodes in the LBM and the time-varying Lagrangian grids for deformable bodies (Peskin, 2002). Under the assumption of no-slip condition at the interface between solid and fluid in IBM, the Lagrangian nodes of the cell surface *x*_*i*_ *(t)* exert forces *F*_*i*_ *(t)* on neighbouring Eulerian nodes *X* on the following equation:

$$f\left(X,t\right)=\sum_{i}F_{i}\left(t\right)\;\delta \left(X-x_{i}\left(t\right)\right),$$ (9)

where $\delta \left(X-x_{i}\left(t\right)\right)$ denotes the discrete Dirac delta function. Subsequently, the Eulerian framework is utilized to calculate the updated velocity and position of the node of the cell membrane, as described by the following Equation.

$$u_{i}\left(t+\delta t\right)=\sum_{i}u_{i}\left(X,t+\delta t\right)\;\delta \left(X-x_{i}\left(t\right)\right),$$ (10)

$$x_{i}\left(t+\delta t\right)=x_{i}\left(t\right)+u_{i}\left(t+\delta t\right)\delta t.$$ (11)

The Dirac delta function is expressed as $=\phi \left(x\right)\phi \left(y\right)\phi \left(z\right)$ , with ϕ representing the one-dimensional interpolation of the kernel function. The formulation of the kernel function is given by:

$$\phi \left(r\right)=\left\{\begin{array}{l} 1-\left|r\right|,\left|r\right|\leq 1\\ 0,\left|r\right|>1 \end{array}\right.$$ (12)

The comprehensive formulation and descriptions regarding the IBM can be found in the earlier studies conducted by Mountrakis et al. (2014; Mountrakis et al., 2015).

### 2.4 Receptor-ligand adhesive interaction model

Our computational model adopts the adhesive dynamics approach developed by Hammer and Apte (1992) to simulate interactions between CTC and endothelial cells. The adhesive dynamics approach by Hammer and Apte (1992) is renowned for studying cell interactions with ligand-coated surfaces, particularly in tumor cell metastasis and adhesion to endothelial cells, as well as in inflammatory responses and lymphocyte homing. Its widespread adoption by previous researchers further validates its suitability for modeling CTC adhesion (Xiao et al., 2017; Zhang et al., 2018; Lenarda et al., 2019; Dabagh et al., 2020; Cui et al., 2021; Wang et al., 2021; Xiao et al., 2021). This model is characterized by a spring-based mechanism with a probability distribution function that governs the adhesion of receptor-ligand pairs, employing a stochastic Monte Carlo technique and integrating a distance-dependent kinetics model as first delineated in the Dembo model (Dembo et al., 1988; Dembo et al., 1988). Dembo model draws an analogy to the “peel test” commonly used in industrial applications. Adhesive bonds in this model are categorized as either “catch” or “slip” bonds, with catch bonds strengthening under tension and slip bonds weakening. In summary, our computational approach integrates these models to simulate cell-wall interactions, contributing to a better understanding of adhesion dynamics in hemostasis.

For adhesive dynamic simulations, the probabilities of bond formation ( $P_{f}$ ) and rupture ( $P_{r}$ ) over a given simulation timestep δt are calculated as using the formulas $P_{f}=1-\exp\left(-k_{f}\delta t\right)$ and $P_{r}=1-\exp\left(-k_{r}\delta t\right)$ , respectively. These probabilities are then compared to a random number drawn from a uniform distribution between 0 and 1 to determine whether a bond is formed or broken. The kinetics of the receptor-ligand bonding process, including both the forward and reverse rates, introduced by Dembo et al. (Dembo et al., 1988) and Hammer and Apte (1992), are governed by the following equations involving parameters such as the equilibrium bond length ( $l_{0}$ ), stretched bond length ( l ), spring constants ( $\sigma_{ts}$ and $\sigma_{b}$ ), Boltzmann constant ( $K_{B}$ ) and absolute temperature ( T ).

$$k_{f}^{n}=k_{f}^{0}\exp\left[-\frac{\sigma_{ts}{\left(l-l_{0}\right)}^{2}}{2K_{B}T}\right],$$ (13)

$$k_{r}^{n}=k_{r}^{0}\exp\left[-\frac{\left({\sigma_{b}-\sigma }_{ts}\right){\left(l-l_{0}\right)}^{2}}{2K_{B}T}\right],$$ (14)

where $k_{f}^{0}$ and $k_{r}^{0}$ are the forward and reverse reaction rates, respectively (Dembo et al., 1988; Hammer and Apte, 1992). The parameter values applied within this study are detailed in Supplementary Table S1, found in the Supplementary Material.

The receptor-ligand bond adhesion force is computed utilizing the spring model, which is delineated as follows.

$${\vec{F}}_{bond}=\sigma_{b}\left(l-l_{0}\right)\vec{e},$$ (15)

where $\vec{e}$ represents the direction of the adhesion force for each bond.

In this study, the adhesion dynamics between CTC and endothelial cells were investigated. The modification to the Dembo model incorporates the influence of wall shear stress on bond association and rupture rates. The wall shear stress enhanced the activation of ligands on endothelial cells, leading to an increased association rate ( $k_{f}$ ) and a decreased rupture rate ( $k_{r}$ ). The modified equations proposed by Yan et al. (2012) are represented as:

$$k_{f}=k_{f}^{n}{\left(\tau /\tau_{0}\right)}^{P_{on}},$$ (16)

$$k_{r}=k_{r}^{n}{\left(\tau /\tau_{0}\right)}^{P_{off}},$$ (17)

where τ is the wall shear stress, $\tau_{0}$ is a reference shear stress, and $P_{on}$ and $P_{off}$ are additional parameters in the model. In our simulation, we defined $P_{on}=1$ , and $P_{off}=-3$ . with a sensitivity analysis conducted on the latter parameter.

### 2.5 Simulation setup and conditions

The simulation setup included a computational domain designed to represent a curved vessel, defined by a cosine function ( $a\left(\cos\left(\pi \left(x-c\right)/b\right)-1\right)$ ), where the curvature amplitude a , determines the TI (the ratio of the curve length along the vessel’s centerline to the linear distance between the two endpoints). Additionally, the curved vessel had a diameter of 20 μm, with each period stretching a distance of 125.6 μm. To build the curved vessel, two straight vessels each with the length of 20 μm were attached to the inlet and outlet of the cosine vessel portion, described above. This curved vessel shown in Figure 4B contrasts with the straight vessel possessing a diameter of 20 μm and a length of 165 μm.

The model incorporates three distinct time steps: (1) *δt*_*Solid*_ for deformable bodies (around 4000 equations per body), (2) *δt*_*LBM*_ for blood plasma flow, and (3) *δt*_*IBM*_ for fluid-solid coupling. Given large deformations and strong adhesion forces, all time steps are set equal to the smallest (0.1 µs). We continued the simulation till the CTC fully passed the curvature shown in Figure 4B as the computational domain with 20 µm diameter, periodic boundaries between inlet and outlet, and smooth no-slip vessel walls by applying the bounce-back algorithm (Lenarda et al., 2019), which effectively simulates the interaction between fluid particles and wall surfaces, ensuring that fluid velocity is zero at the vessel walls. The domain was discretized into a lattice with units measuring 0.5 µm in each spatial direction. Additionally, the flow within the domain was driven initially by a constant body force density applied at each lattice unit, serving as a substitute for the pressure gradient. This force density was adjusted during the initial conditions phase to achieve the desired mass flow rate or maximum velocity, thereby satisfying the assumed shear rate in the straight section of the domain (Kim and Pitsch, 2007). The magnitude of this body force density was directly related to the Reynolds number, which defined the flow rate within the simulated domain. Therefore, a Poiseuille flow condition with a Reynolds number of 0.037 was established as the initial condition of the simulation to satisfy the average shear rate ( $\dot{\gamma }=U_{\max}/D$ ) of 200, a value within the physiological range (Shahidi et al., 2010; Jiang et al., 2014; Wang et al., 2016). The simulations were performed on the Niagara supercomputer of Compute Canada, utilizing 20 Intel Skylake cores (2.4GHz, AVX512) and ran for approximately 45 h to enable the cell center to traverse the entire domain. As this research encompassed multiple domains, each influenced by varying TIs affecting the path length of the cell, the simulation duration might extend to 75 h. Before initiating the main simulations to track cell motion, a warm-up simulation was conducted to ensure that the blood flow reached a fully developed state, where the velocity error was less than 10^–6^ as the convergence criteria (Figure 1).

## 3 Results

### 3.1 Validation of cell motion and adhesion in microcirculations

**FIGURE 2:**
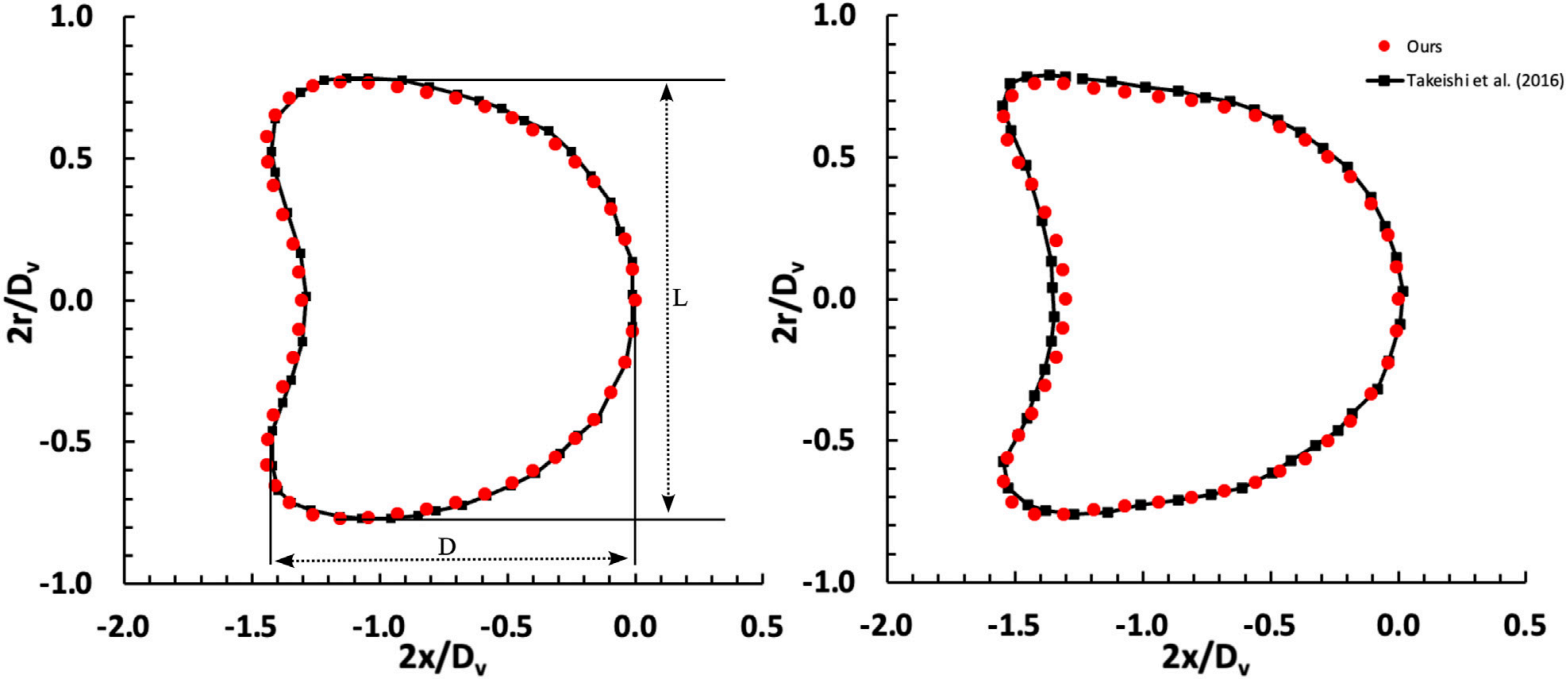
Comparison of deformation of a capsule freely flowing in a capillary between our results and Takeishi et al. (2016). The investigation examines two distinct shear rate values, denoted as capillary numbers: Ca = 0.02 (left) and Ca = 0.052 (right), considering a D_c_/D_v_ ratio of 0.77.

In this section, we assess the accuracy of the employed model by comparing our simulation outcomes with those obtained by Takeishi et al. (2016). They utilized the finite element method to simulate the movement of a capsule within a capillary, a known accurate approach for studying capsule behaviour. To ensure the fidelity of our simulation, we precisely replicated their model and conducted a thorough comparison of our results with theirs. Figure 2 illustrates the changes in the shape of the capsule under various shear rates, demonstrating a close resemblance to the shapes observed by Takeishi et al. (2016). The degree of cell deformability within microchannels can be quantified by the ratio of the major axis length (D) to the minor axis length (L), as cells often deform into a parachute-like shape. At a capillary number (Ca) of 0.052, the deformability ratio (DR = D/L) derived from the results of Takeishi et al. (2016) is 1.0115, whereas our measurement is slightly higher at 1.0137, representing a minimal error of 0.21%. When Ca is reduced to 0.02, the deformability ratio calculated from the results of Takeishi et al. (2016) is 0.937. In contrast, our observed ratio is 0.907 with an error of 3.1%.

To ensure the reliability of our adhesion model and gain a comprehensive understanding of the adhesion behaviour, we investigated the adhesive interactions involving a deformable capsule. Our simulation results were cross-referenced with studies conducted by Zhang et al. (2018) and Wang et al. (2021). The capsule, featuring a spherical morphology with a radius of *R* = 3.75 μm, was placed within a cubic tube measuring *10R × 6R × 6R*. Applying a Couette flow, the top wall of the tube was consistently moved at a shear rate. The fluid properties included a density of *ρ* = 10^3^ kg/m³ and a viscosity of *η* = 10⁻³ Pa·s, resulting in a Reynolds number of Re = 0.1. Capillary numbers (*Ca = ρvRγ˙/K_S_*) were set at 0.005 and 0.015. The dimensionless bond strength (*K*_sp_ = σ_b_/*ρvRγ˙*) was maintained at a constant value of 250 for all simulations. Formation strength (*σ*_*ts*_) was defined as 0.02*σ*_*b*_, while bond rupture strength (*σ*_*b*_ *- σ*_*ts*_) was determined as 0.98*σ*_*b*_. The unstressed formation and rupture rates were influenced by dimensionless parameters (on rate *K*_on_ = $k_{f}^{0}$ /*γ˙* and off rate *K*_off_ = $k_{r}^{0}$ /*γ˙*), mirroring the values employed by Zhang et al. (2018) and Wang et al. (2021).

**FIGURE 3:**
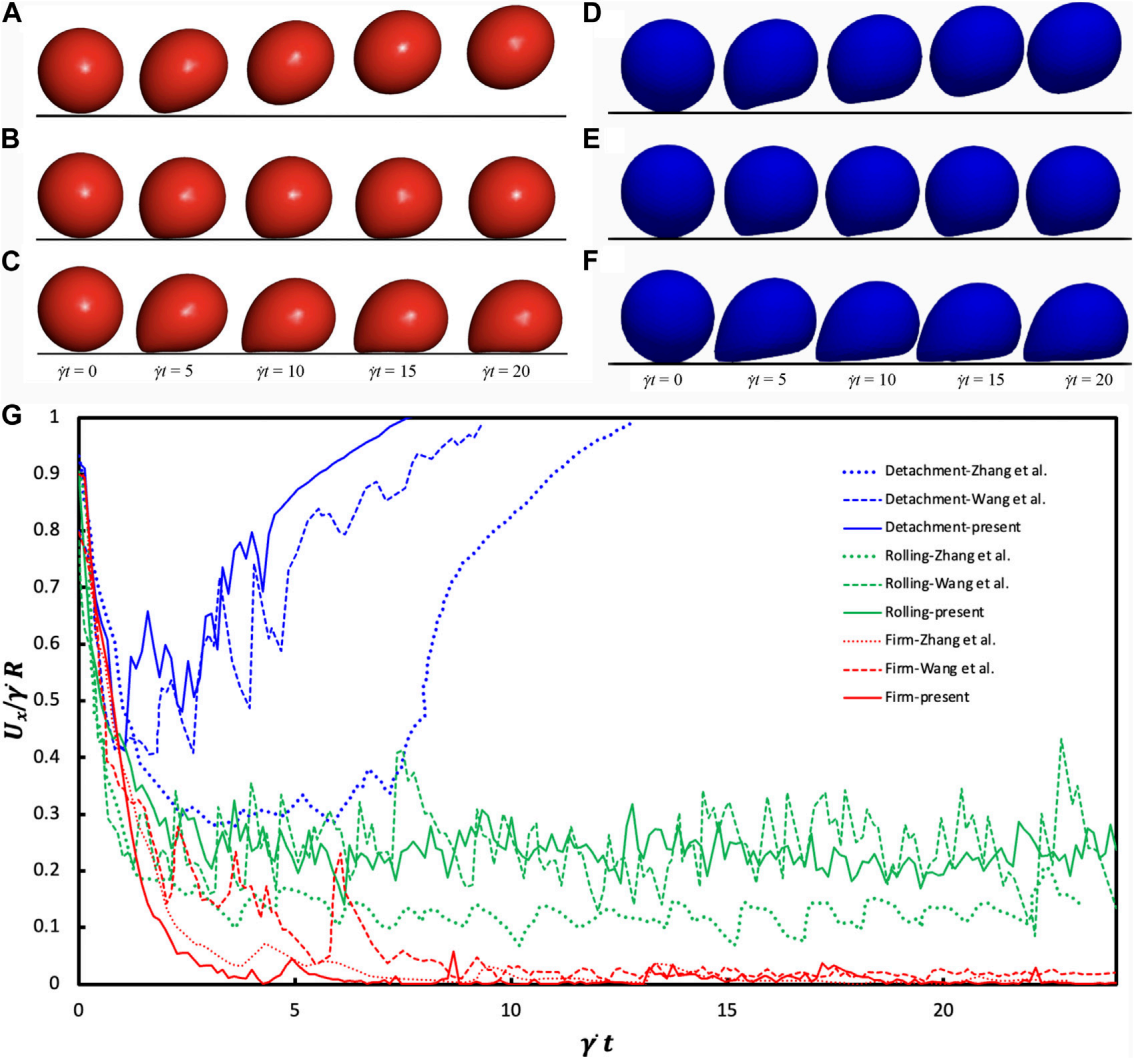
Comparative Analysis of Adhesion States: **(A–C)** findings obtained from Wang et al. (2021) under CC-BY 4.0: https://creativecommons.org/licenses/by/4.0/ license, **(D–F)** from our findings for the last 4 times, Re = 0.1 scenario. The detachment state **(A and D)** emerges under Ca = 0.015 and K_off_ = 1.0 conditions. The rolling adhesion state **(B and E)** is observed at Ca = 0.005 and K_off_ = 0.01. Meanwhile, the firm adhesion state **(C and F)** is pronounced at Ca = 0.015 and K_off_ = 0.01. **(G)** Temporal Changes in Translational Velocity observed across the three adhesion states. The Reynolds number is equal to 0.1, while *K*_on_ remains consistent at 10. Detachment transpires under Ca = 0.015 with *K*_off_ = 1; rolling adhesion manifests at Ca = 0.005 with *K*_off_ = 0.01; and firm adhesion prevails at Ca = 0.015 with *K*_off_ = 0.01.

Figure 3 illustrates three distinct adhesion states within a scenario of Re = 0.1. At a high deformability (*Ca* = 0.015) and rupture rate (*K*_*off*_ = 1.0), the capsule completely detaches, termed “detachment adhesion” (Figures 3A, D). While small deformability (*Ca* = 0.005) and low rupture rate (*K*_*off*_ = 1.0) results in rolling adhesion (Figures 3B, E) lower stiffness (*Ca* = 0.015) leads to firm adhesion (Figures 3C, F). These states closely parallel the findings by Wang et al. (2021).

Figure 3G shows the temporal evolution of the translational velocity across adhesion states. In detachment adhesion, initial bond formation quickly dissociates, resulting in a decreasing then increasing translational velocity. Firm adhesion yields a roughly zero translational velocity due to steady-state bond formation. Rolling adhesion manifests a translational velocity oscillating around a constant value. The disparity in the cell translational velocity between our work and Zhang et al. (2018) and Wang et al. (2021) arises from different deformation models. Our coarse-grained model exhibits strain hardening, presenting a more rigid capsule appearance and higher translational velocity while Zhang et al. (2018) exhibits a softer capsule.

### 3.2 Wall shear stress variations in curved vessels slows down the CTCs and increases extravasation potential

**FIGURE 4:**
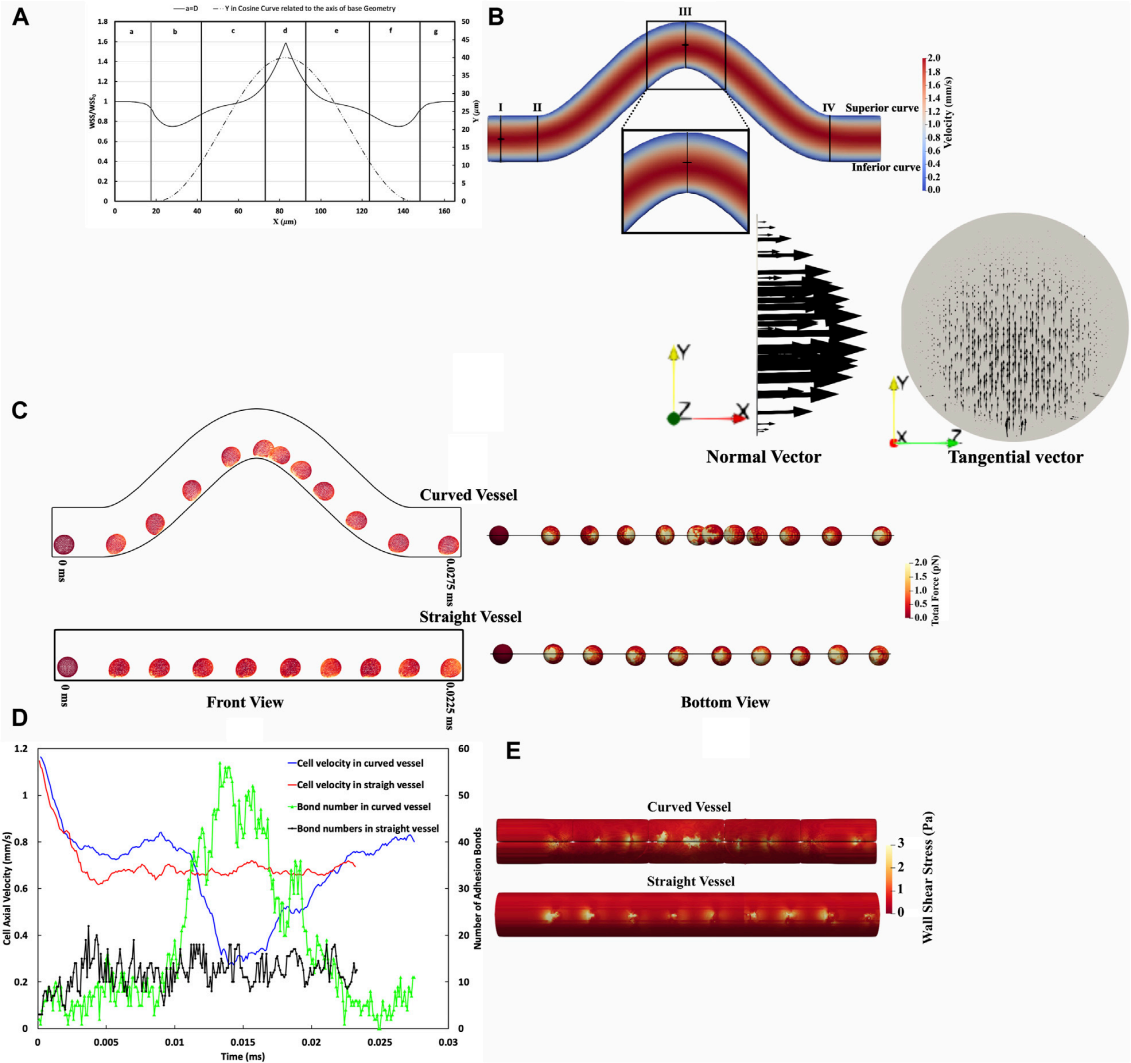
Comparative Analysis of CTC Adhesion and Rolling in Curved Versus Straight Vessels. **(A)** Spatial distribution WSS ratio on the inferior curvature in the curved vessel relative to a straight one considering pure blood flow without any cell with the same Reynolds number. **(B)** The velocity contour dynamics in a curved vessel. The maximum velocity in the straight part (Section aa) occurs at the midpoint of this section, whereas, in the high shear stress region (Section cc), the maximum velocity is skewed toward the inferior curve. **(C)** Temporal sequence of CTC transiting through curved vessel and straight vessel every 0.0025 ms, front view (right), and bottom view (left). These temporal sequences highlight variations in CTC deformation and experience force. **(D)** The temporal variation of CTC velocity and adhesion bonds in vascular geometries, illustrating the differences in CTC velocity and the quantity of adhesion bonds as cells traverse through both curved and straight vessels. In the straight vessel, cell velocity and adhesion bonds are comparatively uniform, in contrast to the curved vessel, where these parameters fluctuate in response to the variable WSS, as indicated in panel **(A)**. **(E)** The WSS distribution in the vicinity of CTCs, illustrating uniform WSS in the straight vessel and variable WSS in the curved vessel, correlating with regions of high and low cell adhesion. It is worth mentioning that the visible lines in the curved vessel contour represent a visualization artifact resulting from the merging of data processed by multiple processors in ParaView.

Our computational model showed the wall shear stress (WSS) in the curved vessel changes over the curvature, causing the cell adhesion and migration to change along the curved vessel. A comparison of hemodynamic and cellular interaction parameters within curved *versus* straight blood vessels is shown in Figure 4. Figure 4A represents the WSS along the x-direction on the inferior outer wall of a cosine-curved vessel, normalized to the WSS obtained in a straight vessel of the same projected length in the x-direction and with the same mass flow rate. The x-axis represents the longitudinal position along the vessel. In the curved vessel, there is a noticeable increase (by 58.5%) in the WSS in regions where the curvature is at its maximum (x = 82.8 μm located at region IV in Figure 4A). This observation is consistent with the general expectation about the spatial evolution of the flow as it progresses within a curvature changing vessel morphology (Balogh and Bagchi, 2019). Conversely, the regions at the start (x = 28 μm located at region II in Figure 4A) and end (x = 137.5 μm located at region VI in Figure 4A) of the curve show decreased WSS by approximately 25%. This pattern suggests alterations in the flow dynamics due to the curvature.

The velocity contour in the curved vessel as illustrated in Figure 4B, shows that the maximum velocity in section cc is not centrally located, unlike the observation in the straight vessel (section aa). Instead, the maximum velocity is skewed toward the inferior curve at the peak of curvature.

In addition, the Dean number (De) is a dimensionless parameter utilized to determine the impact of curvature on the dynamics of fluid flow, characterizing the ratio of centrifugal force to viscous force. It is expressed as $De=Re\sqrt{R/R_{c}}$ (Helin et al., 2009), where $R_{c}$ represents the radius of curvature along the microvessel centerline. The radius of curvature at any point of x for the curve $y=f\left(x\right)$ is given by:

$$R_{c}=\frac{{\left[1+{\left(\frac{dy}{dx}\right)}^{2}\right]}^{\frac{3}{2}}}{\left|\frac{d^{2}y}{dx^{2}}\right|}.$$ (18)

In our study, the Dean number is significantly less than 1, indicating that viscous forces predominate, preventing the development of secondary flow, as demonstrated by the tangential velocity vectors in cross-section cc in Figure 4B.

Investigations into cell dynamics along curved and straight vascular configurations can provide valuable perspectives on the biomechanical variations experienced by cells in these distinct geometries. Figure 4C illustrates that a CTC traversing the curved vessel undergoes notable deformations and experiences fluctuating magnitudes of force over time, as opposed to the more uniform force distribution observed in the straight vessel. Specifically, in the low shear regions II and VI, the total force is 50.41 ± 3.71 pN and 51.15 ± 3.35 pN, respectively, while in the high shear region IV, it is 195.98 ± 9.43 pN. In contrast, in a straight vessel, the force magnitude was almost constant at 75.41 ± 1.99 pN. Furthermore, the comparison of total forces in both geometries highlights the differential mechanical stimuli that cells encounter, which may have downstream effects on cellular adhesion and signalling.

Concerning the adhesion force, the straight vessel exhibits lower variation with an adhesion force of 92.52 ± 2.8534 pN, suggesting a more stable adhesion dynamic. In the curved vessel, however, the total adhesion forces is regulated by the local shear stresses and they decrease in the low-shear regions (61.52 ± 5.22 pN in region II and 63.79 ± 5.71 pN in region VI) and increase in the high-shear regions, showing an adhesion force of 161.36 ± 8.76 pN in region IV. Furthermore, statistical analysis reveals a substantial correlation between the number of adhesion bonds and the overall adhesion force, as evidenced by a correlation coefficient of 77.7%. Furthermore, the effective surface area, over which ligands on the CTC engage during adhesion, is correlated with the number of adhesion bonds. As depicted in Figure 4C, the effective surface area and total number of adhesion bonds in a straight vessel have lower variations than those in the curved vessel. Within the straight vessel, as Supplementary Figure S2 illustrates, the number of adhesion bonds fluctuates between 5 and 16, with a median value of approximately 12. This again indicates a more uniform adhesion process compared to the curved vessel, where the number of bonds varies more remarkably due to shear stress differences. For instance, in the high shear region IV (as shown in Figure 4A), the median number of adhesion bonds for CTCs reaches approximately 40, which is substantially greater compared to the 5 and 6 bonds found in the low shear regions II and VI, respectively.

We studied the effects of vessel configuration on the CTC deformation and quantified these effects using Taylor’s deformation parameter of aspect ratio (Taylor, 1934). Taylor’s aspect ratio, a dimensionless parameter, is calculated by the formula $\varsigma =\frac{L-B}{L+B}$ ; where L and B represent major and minor axes, respectively, of an ellipsoidal CTC. This parameter, ς , was initially described to measure slight deviations from a spherical shape, typically occurring at low flow velocities like those found in plasma moving through microvessels. A value of 0 for Taylor’s aspect ratio indicates a perfectly spherical shape. In the straight vessel, shown in Figure 4C, the cellular deformation appears constant, identified by the Taylor’s aspect ratio of 0.075 in Supplementary Figure S2, during the process of rolling and indicates a quasi-steady state mechanical behaviour during its passage and rolling inside the straight vessel. On the other hand, the Taylor’s aspect ratio of cells in the curved vessel demonstrates significant variations, with a more pronounced deformation in regions of high shear stress (region IV) compared to regions of low shear stress (regions II and VI). As illustrated in Supplementary Figure S2, the aspect ratio of CTCs in the high shear region, specifically region IV of Figure 4A, has the approximate median of 0.11. In contrast, in the low shear regions II and VI, the median of Taylor’s aspect ratio is observed to be around 0.07. This spatial variation in shear stress causes differential cellular deformation, highlighting the intricate interplay between biomechanical forces and cellular morphology during transit in microvessels.

The traversal path within the curved vessel (axis length of 187.15 µm) is elongated compared to the straight vessel (160 µm), leading to prolonged transit times (0.0275 ms in the curved vessel vs 0.0225 ms in the straight vessel). Despite the increased distance, the actual timing for cells to pass through the curved domain varies due to the differential cell velocities encountered in regions of low and high shear stress (Figure 4C).

In Figure 4D, the axial velocity, and the number of adhesion bonds of the CTC traversing both curved and straight vessel geometries are quantitatively presented. It is important to note that due to the stochastic nature of bond rupture and formation, the cell velocity magnitudes exhibited considerable fluctuations. To better illustrate the underlying trends in the cell behaviour and minimize the impact of stochastic variations, we smoothed the data, thereby enhancing the clarity of the observed patterns. The CTC within the curved vessel demonstrates a velocity magnitude that varies substantially along the vessel (maximum axial velocity in the low-shear region = 0.84 mm/s vs minimum axial velocity in the high-shear region = 0.29 mm/s). This spatially-varying velocity magnitude is a direct result of the spatially-varying shear forces inherent in the curved geometry. The cell passing through the straight vessel, on the other hand, has a roughly constant axial velocity with a magnitude of 0.68 ± 0.0069 mm/s.

Figure 4D also illustrates an inverse correlation of −86% between the axial velocity of the CTC in curved vessel and the number of molecular bonds formed between the cells and the vessel wall. Specifically, as the CTC slows down to a minimum axial velocity of 0.27 mm/s between t = 0.014 ms and t = 0.017 ms, it forms up to 56 bonds, creating a favorable condition for forming firm adhesion with the vessel wall.

Furthermore, the comparison of cellular velocities at an entirely straight vessel and the terminal region (post-curvature straight section, region VII) of the curved vessel domain, which approximates the conditions of a straight vessel, reveals notable distinctions (Figure 4D). The CTC transverses faster with an average axial velocity of 0.8 mm/s in region VII compared to an entirely straight vessel with an average axial velocity of 0.68 mm/s along the vessel. This elevation in the velocity magnitude is a consequence of the rupture of bonds and decreased cellular attachments, with 6 adhesion bonds in the low shear region at the end of the curvature (region VI), contrasting with the median number of adhesion bonds of 12 in the straight vessel, as shown in Supplementary Figure S2.

Finally, the presence of cells in close proximity to the vessel wall alters the temporal WSS distribution as shown in Figure 4E. In the straight vessel, the CTCs exhibit a uniform rolling behaviour and velocity, which leads to a consistent and uniform WSS distribution along the vessel wall, a tight maximum WSS range from 6.95 to 7.97 Pa. This uniformity in the cell behaviour and mechanical stress distribution suggests a stable interaction between the cells and the endothelium. Conversely, the adherence of cells to the vessel wall in the curved regions appears to modulate wall shear stress differently. Notably, the maximum WSS in curved vessels shows a broad spectrum, from 4.53 Pa in the low shear regions II and VI at t = 0.0025 ms to 37.52 Pa in the high shear region IV at t = 0.0125 ms. This indicates that the cellular interaction with the endothelium is an important factor in the local hemodynamic environment, with the temporal WSS distribution in the curved vessel being dependent on the position of the CTC along the vessel. Regions II and VI with higher CTC velocities, specifically a median value of 0.78 mm/s, and experiencing lower shear stress, are characterized by a reduced number of stable bonds, around 5 to 6 as shown in Supplementary Figure S2. In these regions, the peak and alteration in the WSS occur in a smaller area due to the reduced interaction and adherence of the cells to the vessel wall. Furthermore, when the CTC passes through regions of high shear stress (region IV), it experiences a decrease in velocity to approximately 0.29 mm/s. Concurrently, there is an increase in the number of cellular bonds to approximately 40.

**FIGURE 5:**
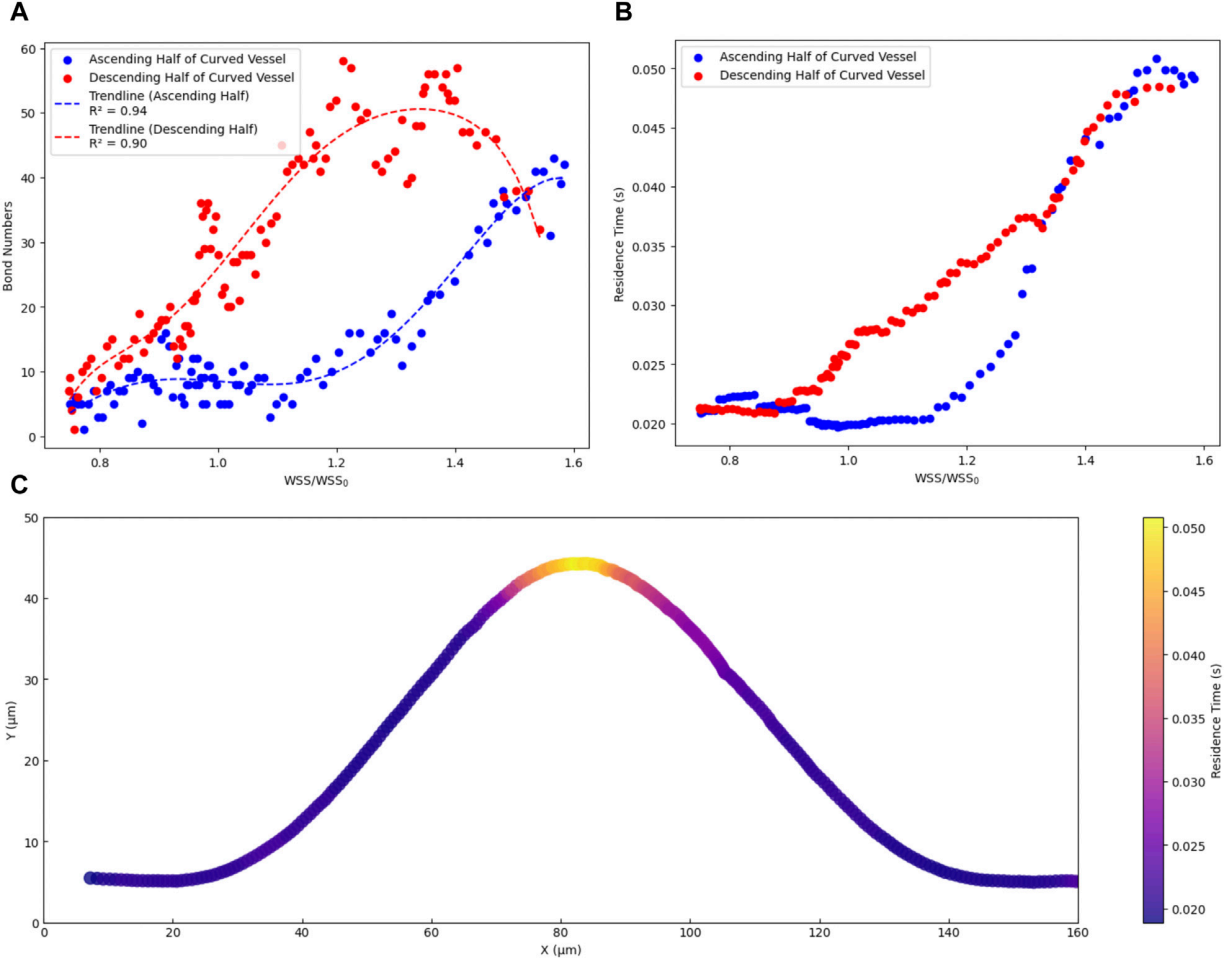
Dependency of dynamics of CTC on Wall shear stress in curved vascular configurations. **(A)** The relationship between the number of adhesion bonds and the WSS ratio. **(B)** The relationship between cell residence time and WSS ratio. **(C)** the trajectory of cells in the x-y plane, with colours representing the residence time of cells within the curved vascular domain.

Furthermore, we conducted a more detailed investigation into the relationship between WSS ratio and the adhesion properties of CTC with a specific emphasis on the complex behaviour displayed by CTCs as they move through curved blood vessels. Figure 5A shows the impact of WSS on the adhesion properties of CTCs, primarily focusing on the number of adhesion bonds formed between the cells and the vessel wall. Notably, in the ascending half of the curved vessel, a strong polynomial relationship was observed between the WSS ratio and the number of adhesion bonds, with an R^2^ value of 0.94. However, as the cell progressed toward the peak of the curved vessel, where WSS reached its maximum, an interesting phenomenon becomes evident. Although the WSS gradually decreased, the number of adhesion bonds initially increased before decreasing. This increase was not solely attributed to the WSS; rather, it was influenced by multiple factors. Particularly in this region (region IV shown in Figure 4A), where the bond rupture rate was low enough, the previously-formed bonds maintained cell adhesion while new bonds continued to form. Afterward, as the influence of decreasing WSS became more pronounced, the number of bonds decreased. Therefore, in the descending half of the curved vessel, a comparable trend between the number of bonds and WSS ratio was anticipated and observed, with one notable exception: the region with the highest WSS (region IV), where the R^2^ value reached to approximately 90%. In addition, investigating the correlation across the entire domain (comprising both the ascending and descending halves of the curved vessel), our analysis revealed a significant Pearson product-moment correlation of 0.72 between the WSS ratio and the number of adhesion bonds. This comprehensive examination highlights the influence of WSS on cellular adhesion dynamics throughout the curved vessel.

Moreover, when a CTC remains in contact with the endothelium with a sufficiently long residence time, a series of interactions occurs leading to the formation of adhesive bonds and subsequent extravasation (Pepona et al., 2020). We conducted a detailed analysis to elucidate the relationship between WSS and the residence time of CTCs on the vascular wall. The residence time was determined by calculating the average duration a cell spent within a specific location along its trajectory. This calculation involved dividing the specific distance a cell travelled (approximately 16 μm, equal to 1 cell diameter) by its time-average velocity within that segment. By repeating this process for various points along the cell trajectory path, an estimate of the time it remained within each location was obtained. Figure 5B demonstrates the dependency of the cell residence time on the WSS ratio. In the ascending half of the curved vessel, an increase in the WSS ratio from 1 to the highest magnitude led to an increase in the cell residence time to 0.05 s. Conversely, within the low shear region (region II in Figure 4A), bond rupture and accelerated cell movement reduced the residence time after this region to 0.02. In the descending half of the curved vessel, post the peak region, the sufficiently high residence time led to new bond formation and decelerating the cell as discussed previously. Furthermore, Figure 5C provides a visual representation of the cell trajectory and associated residence time within the range of 0.02–0.05, offering further insights into the likelihood of optimal locations for cell extravasation.

### 3.3 Wall shear stress modulation with power parameter ( $P_{off}$ ) affects CTC firm adhesion

**FIGURE 6:**
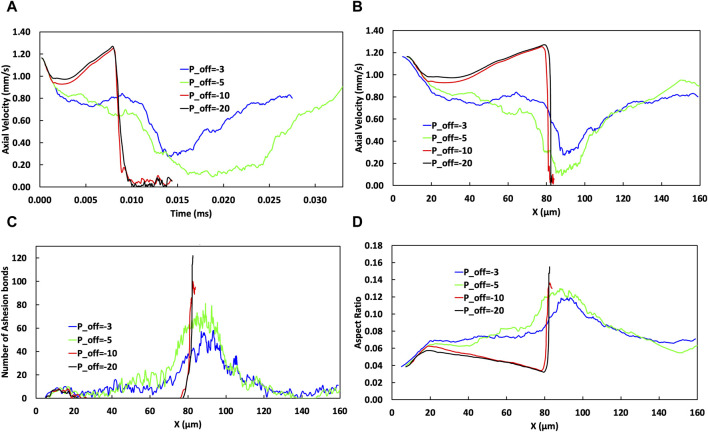
The effect of varying $P_{off}$ on cellular behavior in terms of axial cell velocity, bond numbers, and aspect ratio. **(A–B)** The temporal and spatial evolutions of cell axial velocity. Increasing $P_{off}$ increases the cell axial velocity in low-shear regions and reduces it in the high-shear region, influencing the likelihood of cell detachment in low-shear regions and subsequent reattachment in the high-shear region, particularly at higher $P_{off}$ values of −10 and −20. **(C)** the total number of adhesion bonds as cells traverse the curved vessel, showing that an increase in $P_{off}$ leads to fewer bonds in low shear regions (regions II and VI in Figure 4A) due to elevated rupture rates, while in the high shear region (region IV in Figure 4A), bond numbers increase, potentially leading to firm adhesion when $P_{off}$ exceeds an absolute value of 10. **(D)** The spatial variation in cell aspect ratio, reflecting cell deformation in response to bond dynamics and shear. An increased deformability is observed in the high shear region (region IV in Figure 4A) and reduced deformation in the low shear regions (regions II and VI in Figure 4A) with increasing $P_{off}$ ; in cases of detachment, the cell deforms back, indicated by a decreasing aspect ratio toward zero to recover a spherical shape.

We investigated the influence of the wall shear stress on bond rupture as depicted in Figure 6. Generally, the wall shear stress enhanced the activation of receptors, leading to an increased bond formation rate ( $k_{f}$ ) and a decreased bond rupture rate ( $k_{r}$ ). It is important to highlight that the rupture rate determines the time scale of ligand-receptor bindings (Takeishi et al., 2016), was shown to impact the adhesive behaviour of cells and is generally critical for the transition from firm adhesion to detachment, and from rolling adhesion to detachment, as demonstrated in previous studies by Zhang et al. (2018) and Wang et al. (2021) and discussed in the Results.

Figure 6 illustrates how varying the power parameter, $P_{off}$ , in Eq. (17) modulates the effect of shear stress on the rate of bond rupture. Notably, as $P_{off}$ value increases in negativity, there is a marked increase in the bond rupture rate in the low-shear regions ( $\tau /\tau_{0}<1$ ), with the converse effect in the high-shear regions ( $\tau /\tau_{0}>1$ ). Specifically, a decrease in the WSS ratio ( $\tau /\tau_{0}$ ) from 1.00 to 0.75, accompanied by an increase in $P_{off}$ from −1 to −2, results in a 33.3% increase in the rate of bond rupture (not shown in Figure 6). In contrast, with the same change in $P_{off}$ , an elevation of the WSS ratio from 1.00 to 1.25 results in a 20% reduction in the rate of bond rupture (not shown in Figure 6).

The temporal and spatial change of CTC axial velocity is illustrated in Figures 6A,B, respectively. Changing the values of $P_{off}$ results in distinct temporal and spatial velocity evolutions, suggesting that the bond rupture rate has a direct and variable impact on the cell axial velocity over time and in both low and high shear regions. Increasing the value of $P_{off}$ in negativity from −3 to −5 does not lead to cell detachment in regions of low-shear stress (region II), but it does result in increased cell velocity, from 0.792 mm/s to 0.842 mm/s as observed Figure 6B. This change indicates that while bonds are rupturing and the total number of bonds is reduced from mode number 5 to 4 (Figure 6C in the low-shear region II between x = 20–40 µm), the number of ruptures is not sufficient to cause detachment, but enough to alter the cell axial velocity. Conversely, in the high-shear region IV, this increase in the power parameter, although insufficient to promote firm adhesion, results in a decreased rate of rupture. The reduction in the bond rupture rate is particularly significant at the vessel peak (Section cc in Figure 4B), where it decreases by up to 60% in response to a 58.5% increase in the WSS ratio. The decrease in the rupture rate, associated with a shift to more negative $P_{off}$ values from −3 to −5, is associated with the increase in the total number of bonds from a mode of 43–70 (Figure 6C) and a reduced cell velocity from an average 0.3118 ± 0.0038 mm/s to 0.12618 ± 0.0033 mm/s (Figure 6B). This implies that regions where bonds are less likely to rupture enable the cell to maintain a slow velocity closer to the vessel wall, potentially due to the increased strengths of receptors on the endothelial cells. Additionally, the range over which the minimum cell velocity (within ±10%) is observed, extends further, increasing from 8.7 µm to 12.5 µm by a shift to more negative $P_{off}$ values from −3 to −5. This implies that the CTC rolls for a prolonged duration, from 0.0026 ms (associated with the $P_{off}$ value of −3, observed from t = 0.0141 to t = 0.0167 ms as shown in Figure 6A) to 0.0078 ms (associated with $P_{off}=-5$ from t = 0.0165 ms to t = 243 ms) with a minimum axial velocity (varying within ±10%), suggesting more persistent interactions with the vessel wall despite the high shear stress.

Further increasing $P_{off}$ in negativity from −5 to −10 and then −20 causes a significant rise in the bond rupture rate in the low-shear regions (regions II and VI). The bond rupture rate increases by a factor of 4.25 when shifting $P_{off}$ from −5 to −10 where the WSS ratio is at its minimum (WSS ratio of 0.7487 at x = 28 µm shown in Figure 4A), and by 18 times when changing $P_{off}$ from −10 to −20, leading to cell detachment as can be seen in Figure 6C with zero number of bonds around x = 25 µm. Consequently, cells accelerate (Figure 6B) to match the velocity of blood flow, increasing from 0.94 mm/s at ∼25 μm to 1.25 mm/s at ∼78 µm when $P_{off}=-10$ , and from 0.97 mm/s at ∼25 μm to 1.26 mm/s at ∼80 µm when $P_{off}=-20$ . As the detached cell transits through the high-shear regions, such as around x = 75 µm shown in Figure 6C, it is drawn towards the wall by the flow profile observed in Figure 6B, encouraging new bond formation. In the high-shear region around x = 75 μm, there is a remarkable reduction in the rate of bond rupture, with a tenfold decrease when the $P_{off}$ value changes from −5 to −10 and a hundredfold decrease when it changes from −10 to −20. The endurance of previously formed bonds under these conditions allows the cell to establish additional bonds, leading to firm adhesion. This is demonstrated by the near-zero cell velocity just after x = 80 µm and over time as shown in Figures 6A,B.

Figure 6D shows the evolution of the cell aspect ratio as the cell travels through the microvessel. The aspect ratio is a measure of cellular deformation and because it is influenced by the wall shear stress, and its variation along the vessel, it is a marker for the mechanical stress encountered by cells. An increase in negativity in $P_{off}$ from −3 to −5 correlates with a reduction in the number of bonds from mode number 5 to 4 in the low shear regions (Figure 6C between x = 20–40 μm, region II) which in turn results in a decreased aspect ratio from 0.068 to 0.063. However, upon reaching of the high shear stress region IV, the cells exhibit an increase in the aspect ratio from 0.116 to 0.125, indicative of more pronounced deformation due to a greater number of bonds from mode number 43 to 70 (Figure 6C) and a reduced probability of bond rupture.

Increasing the absolute value of $P_{off}$ to 10 and 20 represents a more complicated relationship. Initially, bond formation does occur, causing an increase in the aspect ratio during the initial timesteps. However, the extent of the increase is less than what is observed with $P_{off}$ of −3 and −5, which suggests a lower level of deformation. Specifically, the aspect ratio increases to 0.062 and 0.057 for *P*_*off*_ values of −10 and −20, respectively, compared to the increases of 0.069 and 0.065 for $P_{off}$ values of −3 and −5, respectively. Additionally, as discussed previously, the elevated rate of bond disruption contributes to CTC detachment, leading to a post-detachment transition of cells towards a spherical shape in the free blood flow, which is reflected in a decreased aspect ratio between x ≈ 20 and x ≈ 80 µm shown in Figure 6D. The aspect ratio reduced from 0.057 to 0.032 for $P_{off}=-20$ , and it decreased from 0.062 to 0.034 at $P_{off}=-10$ . Furthermore, at the high shear region close to the curved vessel peak (Section cc in Figure 4B), the cells begin to establish new bonds, resulting in an increase in the deformation and, as a result, an increased aspect ratio. The greatest deformation is found under these firm adhesion conditions, with aspect ratios reaching a high of 0.133 for $P_{off}=-10$ and 0.147 for $P_{off}=-20$ .

### 3.4 Increasing tortuosity index in curved vessels alters cell velocity, adhesion, and deformation for extravasation

The vessel curvature can substantially influence the WSS. Increasing the tortuosity level in a curved vessel by elevating the amplitude ( a ) of the cosine function ( $a\left(\cos\left(\pi \left(x-c\right)/b\right)-1\right)$ ), which defines the vessel curvature, can intensify the maximum WSS in the high-shear region IV. Simultaneously, it can reduce the minimum WSS in the low-shear regions II and VI. Changing the amplitude from *a = D* to *2D* results in a 50% increase in the maximum WSS ratio, and a 15.3% decrease in the minimum WSS ratio. These changes in WSS, shown in Supplementary Figure S3, profoundly affect cellular behaviour within vessels of different TIs.

**FIGURE 7:**
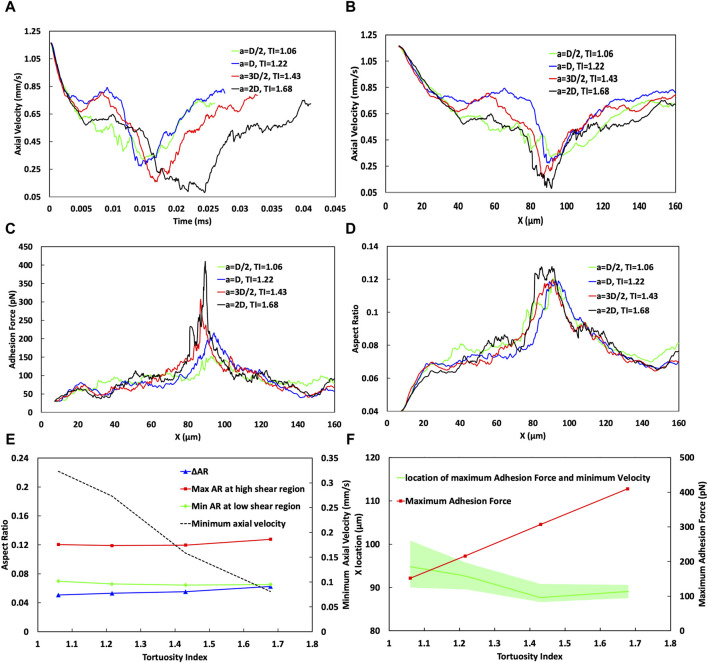
Analysis of the effect of the vessel Tortuosity Index (TI) on cell adhesion dynamics. **(A)** Temporal variation of cell velocity across different TIs, demonstrating the influence of vessel tortuosity on the transit time of CTC through the curved vessels. **(B)** Spatial variation of CTC velocity for varying TIs, showing consistent speeds at the initial straight part (*x* < 20 µm) and divergent behaviours in the low shear region (20–40 µm). In the high shear region, especially near *x* = 90 μm, the velocity substantially decreases with higher TIs, showcasing a trend of reducing minimum velocity as TI increases, which is indicative of stronger cell adhesion in regions of greater curvature. **(C)** Adhesion force mapping along the vessel path, with distinct peaks at high shear region VI or different curve amplitudes, revealing a higher adhesion force and a lower bond rupture rate for vessels of higher TIs. **(D)** Variation in the aspect ratio of CTCs indicative of cell deformation through the curved vessel, comparing different TIs. **(E)** Minimum cell velocity, maximum and minimum cell aspect ratio, and their difference as a function of TI, emphasizing the deceleration of cells and increase in cell deformation at higher TIs. **(F)** Maximum adhesion force as a function of the TI, emphasizing an increase in adhesion forces at higher TIs in the high shear region around *x* = 90 μm, and estimation of the most likely position for maximum cell adhesion and minimum velocity, with a shift towards larger *x* values for lower TIs, but occurring nearer to the peak curvature for higher TIs, suggesting increased chances of cell extravasation due to stronger adhesion.

The effects of the cosine curve amplitude and its corresponding TI on the cell adhesion behaviour is examined in Figure 7. Figure 7A shows that changes in the TI not only change the traverse path length but also influence the behaviour of the CTC within the curved vessel (including the temporal evolution of the axial cell velocity). Consequently, an elevation in the TI is associated with a prolonged transit time through the curvature. Specifically, a TI of 1.68 corresponds to a transit time of 0.0411 ms, while a lower TI of 1.06 results in a shorter transit time of 0.026 ms.

The behaviour of cells under different conditions, such as varying WSS and TIs can be quite complex and can be influenced by a complex interplay between hydrodynamic forces and cellular adhesion dynamics.

In the region where *x* < 30 μm, cells begin to roll along the straight portion of the vessel before reaching the location of the minimum WSS ratio at about *x* = 23 µm in the curved portion near the end of the straight portion, as shown in Supplementary Figure S3. In this region, across all TI values, the axial cell velocity demonstrates consistent behaviour with an error margin of less than 1%, decreasing from 1.2 mm/s at the inlet to 0.75 mm/s at x = 30 µm (Figure 7B). This indicates that, within this specific region, CTCs are not significantly influenced by variations in the vessel geometry, or the shear forces encountered.

However, at x∼40 μm, there is a notable difference in the cellular behaviour by varying TIs. For TI = 1.06, the adhesion force reaches 94.5 pN (Figure 7C) remarkably surpassing the forces at other TIs, which measure 66.3, 67.31, and 68.88 pN for TIs of 1.22, 1.43, and 1.68, respectively. Despite the WSS ratio being below 1 (Supplementary Figure S3), for TI = 1.06 (*a = D/2*) it stands at 0.86, higher than that for TI = 1.22 (*a = D*), which is 0.75. This higher WSS correlates with an 11.4% increase in bond formation rate and a 66.3% reduction in bond rupture rate for TI = 1.06 compared to the baseline (TI = 1.22). Consequently, there is a 40% increase in the adhesion force (comparing 94.5 pN for TI = 1.06–66.3 pN for TI = 1.22). This phenomenon illustrates a situation where even though the WSS is higher, which would typically lead to increased hydrodynamic forces on the cell that could speed up its movement, the significantly larger adhesion force dominates, resulting in a decreased cell velocity by 15.6%, from 0.745 mm/s at TI = 1.22 to 0.629 mm/s at TI = 1.06.

Conversely, at the same location (*x* ∼ 40 μm), when comparing TI = 1.22 to TI = 1.68, the adhesion forces are similar, suggesting that adhesion forces alone do not significantly affect cell velocity. Instead, the decrease in the minimum WSS ratio from 0.75 to 0.634 (as TI increases from 1.22 to 1.68) seems to be the dominant factor that causes the cell velocity at *x* ∼ 40 μm to decrease from 0.745 mm/s to 0.624 mm/s.

Furthermore, one of the most important phases of cell passage occurs around the vessel peak (cross section cc in Figure 7B), in the high shear region IV, specifically close to 90 µm. This phase is marked by the effect of the increased WSS on endothelial cell receptors, resulting in a reduced rate of receptor rupture. Consequently, a maximum adhesion force is reached (409 pN for higher TIs compared to 151 pN for lower TIs as depicted in Figures 7C,F), alongside a minimum of the cell velocity in this zone for all TI values (Figure 7B). The minimum velocities are 0.0809 mm/s for the highest TI (1.68) and 0.32 mm/s for the lowest TI of 1.06 (Figures 7B,E). While an increase in TI close to the curvature peak results in elevating the WSS and shear forces, which are generally expected to increase cellular velocity, it is observed that within the region spanning *x* = 87–97 μm, the adhesion force exerts a more significant influence than the hydrodynamic forces. Furthermore, as TI values increase, the deceleration of cells in high-shear regions becomes increasingly noticeable. This is evident where cells exhibit a notably lower minimum velocity of 0.0809 mm/s with a TI of 1.68, in contrast to the 0.32 mm/s observed with a TI of 1.06 which increases the chance of extravasation for CTC.

The most probable location for CTC extravasation, considering both the minimum velocity and maximum adhesion force, has been predicted while accounting for a range of ±10% around these magnitudes. As illustrated in Figure 7F, this location shifts to downstream (larger *x* values) for lower TIs, for instance, after the peak of the curve at *x* ≈ 94 µm within the range of ∼90–100 µm with TI = 1.06. Conversely, for higher TI (1.68), this event occurs closer to the peak at ∼89.5 µm, within a narrower range of ∼87–90 µm. This smaller range at higher TI correlates with the narrower peak of maximum WSS in Supplementary Figure S3, which, as previously explained, has a significant impact on both adhesion dynamics and cell behaviour.

As cells initiate rolling at the start of their transit through the vessel, their aspect ratio increases to 0.055 in the *x* < 20 μm range, indicating cell deformation and elongation due to the interplay between hydrodynamic forces and adhesive interactions. This deformation is further evident in Figure 7D, by the change in the deformation and aspect ratio of the CTC while passing through the curved vessel of different TIs.

Before *x* < 30 μm, the aspect ratio across all TIs is relatively similar. For example, at TIs 1.06 and 1.22, the aspect ratio is around 0.068, while for TI = 1.43, it starts from 0.069 and decreases to 0.065, and for TI = 1.68, the aspect ratio is equal to 0.064. Thus, the initial deformation across different TIs shows only a slight variation with a maximum difference of 6%. The first notable difference in deformation is observed around *x* ≈ 40 µm. At this location, for TI = 1.06, the adhesion force peaks at 94.5 pN, significantly higher than the forces measured at other TIs—66.3, 67.31, and 68.88 pN for TIs of 1.22, 1.43, and 1.68, respectively. Consequently, due to the higher adhesion force at TI = 1.06, a greater deformation is expected compared to other TIs (0.08 *versus* 0.07, 0.069, and 0.068 for TIs 1.22, 1.43, and 1.68, respectively).

Furthermore, the cells experience their maximum deformation when they migrate into the high shear region (approximately 80–90 µm). Despite the aspect ratio for TI = 1.06 to TI = 1.43 remaining within an approximate range of 0.12, as shown in Figures 7D,E, it escalates to 0.1276 for a TI = 1.68. This trend highlights the greater deformation experienced by cells at higher TIs due to the combined impacts of increased adhesion forces and increased shear force due to the high shear conditions. The comprehensive analysis across the entire vessel passage in Figure 7E reveals a significant trend. The change in the aspect ratio (ΔAR), indicative of the maximum deformation, increases from 0.05 to 0.062 with higher TIs.

### 3.5 Softer cells excel in extravasation when they remain attached in low-shear regions

Cell stiffness, quantified by the shear modulus (*G*) and related to the k_link_ parameter in the computational model as described in Materials and Methods, is an important biomechanical property that influences cellular behaviour, particularly in dynamic environments such as vascular systems where blood flow-induced shear stress is substantial. Katsantonis et al. (1994) proposed that the process of malignant transformation in tumor cells decreased F-actin composition in the cell cytoskeleton, which results in higher deformability of these cancerous cells. The ability of a cell to deform, adhere to surfaces, and detach under shear stress is intrinsically linked to its stiffness as explained in Validation.

Furthermore, we studied the effect of CTCs’ stiffness on their adhesion to the vessel wall in curved vessels. We considered CTCs with the shear modulus ranging from 30 to 270 μN/m, a magnitude that aligns with numerical and experimental studies (Guz et al., 2014; Bagnall et al., 2015; Xiao et al., 2017; Lenarda et al., 2019; Deliorman et al., 2020; Kwon et al., 2020). Numerical simulations by Xiao et al. (2017) and Lenarda et al. (2019) have employed G values ranging from 4.16–416 μN/m and 5–200 μN/m, respectively, to model CTCs with different stiffness levels. Moreover, Lenarda et al. (2019) refer to experimental studies (Guz et al., 2014; Bagnall et al., 2015) suggesting that CTCs can exhibit elasticity values ranging from 1 kPa to 100 kPa, which, considering a membrane thickness of 10 nm (Skalak et al., 1973), translates to approximately 10–1,000 μN/m. Considering an isotropic triangular mesh with a Poisson’s ratio of 1/3, which relates the elasticity modulus (E) to shear modulus (G) as E = 8/3G (Tan, 2015), the corresponding shear modulus values range from 3.75 to 375 μN/m. Experimental investigations using atomic force microscopy (AFM) by Deliorman et al. (2020) reported elasticity values of 6.2 ± 1.8 kPa for soft CTCs and 23.9 ± 2.2 kPa for stiff CTCs, with a maximum of 45 kPa. The corresponding shear modulus values range from approximately 23.25 ± 6.75 μN/m for soft CTCs to 89.625 ± 8.25 μN/m for stiff CTCs, with a maximum value of 168.75 μN/m. Furthermore, Kwon et al. (Kwon et al., 2020) have provided comparative data on the deformability of various cancer cell types, reporting shear modulus values ranging from 17.5–51.4 μN/m for breast cancer cells, 51.85–136.5 μN/m for cervical cancer cell lines, and 38.55–196.46 μN/m for lung cancer cell lines.

**FIGURE 8:**
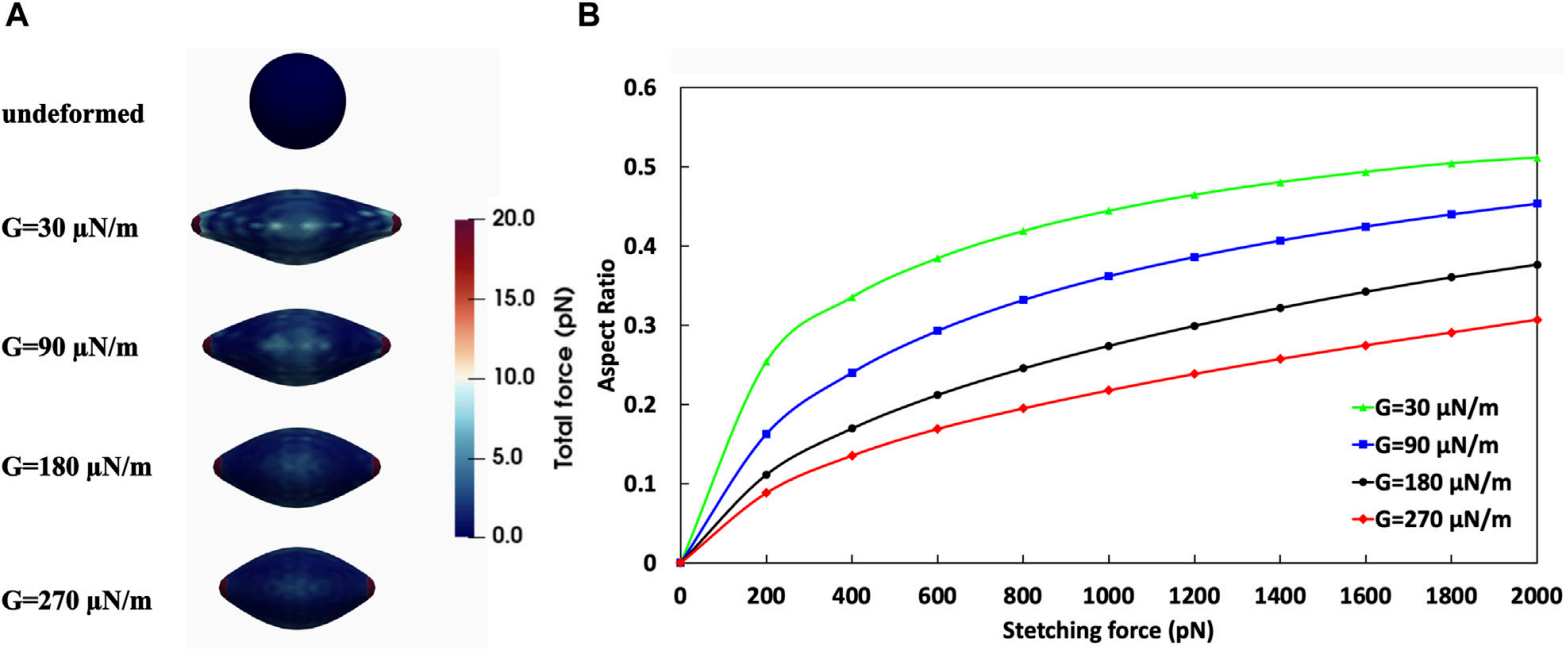
Analysis of the effect of CTC shear moduli on its deformation. **(A)** Stretch test simulation on CTCs with different stiffness. Top: undeformed CTC; Bottom: CTCs with various shear moduli under tension force of 2000 pN; **(B)** Aspect Ratio-Tension Force obtained from numerical simulation of stretch test.

We conducted numerical simulations of stretch tests on CTCs of these various shear moduli employing techniques similar to those used in both experimental (Suresh et al., 2005a) and computational (Fedosov et al., 2010b) studies for red blood cells (RBCs). These numerical stretch test simulations were performed based on the methodology outlined in Section 2, where the *G* values were defined according to the method described in Section 2.2. Given the greater rigidity of CTCs compared to RBCs, the maximum tension force applied was 2000 pN, which is ten times the maximum force typically used in stretching tests for red blood cells. The outcomes of these numerical stretch tests for the different cell stiffness are detailed in Table 1. In Figure 8A, the force distribution on an 8-μm diameter CTC is shown, beginning from an initial state of zero applied force and ending after the stretch test, where the CTC is subjected to a 2000 pN tension force at *G* values of 30, 90, 180, and 270 μN/m. Upon completion of the numerical stretch test, the maximum force that endured locally was 20 pN, with the major and minor axes measuring 17.85 μm and 5.7695 μm, respectively, resulting in an aspect ratio of 0.51 for the most deformable cell (G = 30 μN/m). On the other hand, the stiffest cell (G = 270 μN/m) had an aspect ratio of 0.3 due to its major and minor axes being 12.86 μm and 6.87 μm, respectively. Figure 8B demonstrates the evolution of the aspect ratio for cells of varying stiffnesses throughout the stretch test. This figure indicates that the largest variation in cell deformation, as measured by aspect ratio, between the softest and stiffest CTCs is roughly 70%.

**TABLE 1:** Results of the stretch test of CTCs with different shear moduli.

G (μN/m)	Stretch test (optical tweezer)	Stretching force (pN)										
		0	200	400	600	800	1,000	1,200	1,400	1,600	1800	2000
30	Largest Diameter of CTC (µm)	8	11.797	13.274	14.306	15.113	15.769	16.314	16.773	17.163	17.499	17.850
	Smallest Diameter of CTC (µm)	8	7.018	6.607	6.360	6.191	6.069	5.966	5.886	5.821	5.767	5.770
	Aspect Ratio (ξ=(L-B)/(L + B))	0	0.254	0.335	0.384	0.419	0.444	0.464	0.480	0.493	0.504	0.511
90	Largest Diameter of CTC (µm)	8	10.417	11.568	12.399	13.072	13.649	14.160	14.621	15.041	15.427	15.782
	Smallest Diameter of CTC (µm)	8	7.504	7.090	6.781	6.559	6.395	6.270	6.164	6.077	6.003	5.937
	Aspect Ratio (ξ=(L-B)/(L + B))	0	0.163	0.240	0.293	0.332	0.362	0.386	0.407	0.424	0.440	0.453
180	Largest Diameter of CTC (µm)	8	9.691	10.572	11.232	11.765	12.220	12.624	12.992	13.332	13.651	13.951
	Smallest Diameter of CTC (µm)	8	7.749	7.509	7.305	7.130	6.966	6.811	6.667	6.535	6.418	6.319
	Aspect Ratio (ξ=(L-B)/(L + B))	0	0.111	0.169	0.212	0.245	0.274	0.299	0.322	0.342	0.360	0.377
270	Largest Diameter of CTC (µm)	8	9.351	10.083	10.647	11.113	11.506	11.851	12.161	12.446	12.712	12.964
	Smallest Diameter of CTC (µm)	8	7.836	7.681	7.572	7.492	7.396	7.292	7.188	7.087	6.990	6.878
	Aspect Ratio (ξ=(L-B)/(L + B))	0	0.088	0.135	0.169	0.195	0.217	0.238	0.257	0.274	0.290	0.307

**FIGURE 9:**
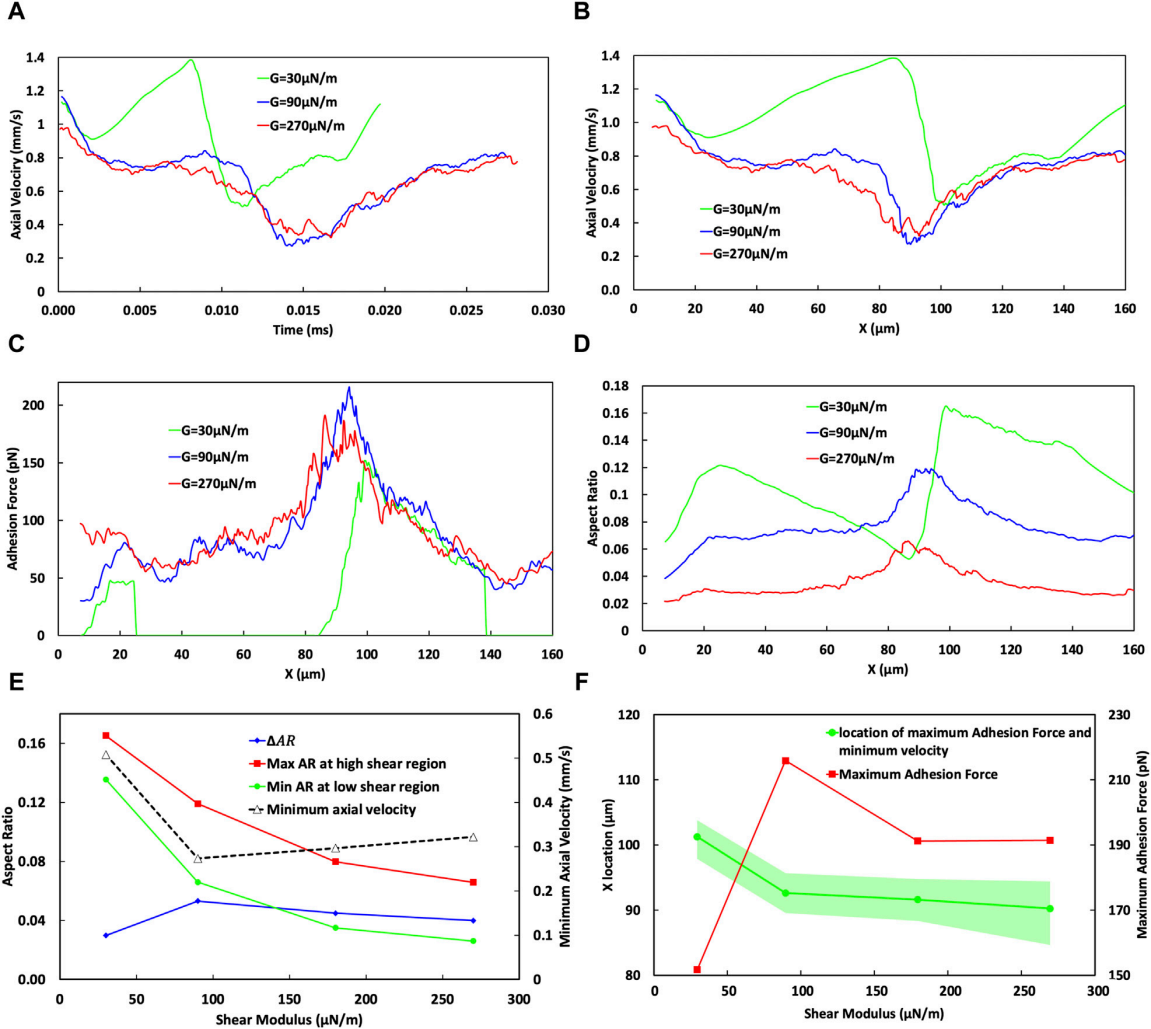
Analysis of the effect of CTC stiffness on its adhesion dynamics. **(A)** Temporal variation of cell velocity across different cell shear moduli, demonstrating the influence of cell stiffness on the transit time of the CTC through the curved vessels. **(B)** Spatial variation of CTC velocity for varying cell stiffness, showing divergent behaviours in the low-shear region (20–40 µm) for varying stiffness specifically for very soft cells, indicating the cell detachment in the low-shear region. **(C)** Adhesion force mapping along the vessel path, with distinct peaks at high-shear regions for cells of different shear moduli, revealing a higher adhesion force and a lower bond rupture rate for softer cells, assuming cell attachment in the low-shear region. **(D)** Variation in the aspect ratio of CTCs indicative of cell deformation through the curved vessel for different cell stiffnesses. **(E)** Minimum cell velocity, maximum and minimum cell aspect ratio, and their difference (ΔAR) as a function of the shear modulus of the cell, emphasizing the deceleration of cells and increase in cell deformation for softer cells, in case of rolling over whole domain **(F)** Maximum adhesion force as a function of the shear modulus of the cell, emphasizing a decrease in adhesion forces for the stiffest CTCs in the high shear region around *x* = 90 μm, suggesting increased chances of cell extravasation due to stronger adhesion; and estimation of the most likely position for maximum cell adhesion and minimum velocity, with a shift towards larger *x* values for softer cells, but occurring nearer to the peak curvature for the stiffest CTCs.

The effects of varying cell stiffness on cell behaviour, with a particular focus on the aspect ratio, velocity, and adhesion forces of cells, are presented comprehensively in Figure 9. This figure examines the relationship between shear modulus (denoted as *G* with values 30, 90, and 270 μN/m) and different biomechanical responses. The temporal (Figure 9A) and spatial (Figure 9B) variations in the axial cell velocity reveal that the most deformable cell (*G* = 30 μN/m) exhibits distinct behavioural dynamics compared to stiffer cells.

The adhesion force variation for the softest cell (green curve in Figure 9C) indicates that following the establishment of bonds and a subsequent reduction in the velocity within the initial straight segment of the vessel, CTC experiences detachment in the low shear region VI. This detachment may be attributed to an increased bond rupture rate and the low stiffness of the cell, as explained in Section 3.1. The following increase in the velocity of the softest CTC after its detachment signifies the free movement of CTC in blood flow. Nevertheless, due to the intrinsic behaviour of blood flow in curved vessels, where the maximum velocity of flow deviates toward the inferior wall, the cell benefits by migrating toward the inferior wall, therefore promoting the opportunity for bond reestablishment.

The aspect ratio of the cell is shown in Figure 9D. Upon detaching (at *x* ≈ 25 µm), the softest cell undergoes a transformation towards a more spherical state, as evidenced by a reduction in the aspect ratio from 0.136 during detachment to 0.052 upon reattachment (at *x* ≈ 85 µm) due to the release of adhesive constraints (Figure 9C). The reduction in the aspect ratio is indicative of the cellular tendency to revert to its initial spherical configuration when no external constraints and forces are present.

As CTC moves into the high-shear region IV and reattaches, the bond rupture rate is reduced, leading to an increased lifetime of the formed bonds. The high deformability of the softest cell enhances its effective area for interaction with the wall, which, in turn, reduces its velocity to 0.5 mm/s and increases its aspect ratio to 0.165 due to the increased adhesion forces shown in Figures 9C,D for *G* = 30 μN/m where the cell is located around the curved vessel peak (cross section cc). However, as the cell begins to roll toward the low-shear stress region VI, the bond rupture rate concurrently increases, which is evidenced by a gradual increase in CTC velocity from a local minimum in the CTC velocity waveform in the second half of the domain at *x* ≈ 100 µm shown in Figure 9B. Consequently, the CTC experiences a concomitant gradual decline in the aspect ratio to 0.135 at *x* ≈ 138 µm immediately before its eventual detachment in the low shear region at the end of the curvature in region VI.

Comparatively, for CTCs that do not detach in the low shear regions, specifically in region IV, with the shear modulus of *G* = 90 *versus G* = 270 μN/m, the softer cell (*G* = 90 μN/m) travels faster due to bond rupture in the low-shear region (0.77 mm/s for *G* = 90 μN/m *versus* 0.71 mm/s for *G* = 270 μN/m, with adhesion forces of 46 *versus* 60 pN, respectively). However, as the CTC with the shear modulus of *G* = 90 μN/m transitions into the high shear region IV, its increased deformability allows for the formation of more bonds. Consequently, the maximum adhesion force observed for the softer cell, assuming the cell does not experience detachment, is greater (216 pN for *G* = 90 μN/m *versus* 191 pN for *G* = 270 μN/m), resulting in a slower velocity (0.27 mm/s for *G* = 90 μN/m *versus* 0.32 mm/s for *G* = 270 μN/m), as shown in Figure 9B, Figure 9E, and Figure 9F. This reduction in the adhesion force and increase in the minimum velocity of the CTC are critical factors as they reduce the likelihood of extravasation, implying that for optimal metastatic potential, a CTC cannot be excessively soft or rigid; there exists an optimal stiffness that maximizes the probability of extravasation.

The elevated aspect ratio of the CTC with *G* = 90 μN/m compared to *G* = 270 μN/m, as illustrated in Figures 9D,E, corresponds to its increased deformation. Specifically, the maximum aspect ratio for *G* = 90 μN/m is 0.12, compared to 0.066 for *G* = 270 μN/m. Similarly, the minimum aspect ratio within the curved section (20 µm < x < 145.6 µm) for *G* = 90 μN/m is 0.066, an increase from the 0.026 observed for *G* = 270 μN/m. The aspect ratio displays similar trends across various stiffness levels, assuming the cell does not experience detachment. In general, as indicated by the change in the aspect ratio (ΔAR = maximum AR–minimum AR) in Figure 9E, an increase in the cell stiffness (in term of the shear modulus) is correlated with a decrease in both overall deformation and aspect ratio. Specifically, increasing the cell stiffness threefold from *G* = 90 μN/m results in a decrease in ΔAR from 0.53 to 0.4.

Furthermore, as the stiffness increases, the optimal extravasation location shifts as illustrated in Figure 9F, where adhesion force and velocity are at their maximum and minimum, respectively. Stiffer cells exhibit a wider spatial distribution and are in closer proximity to the vessel peak, where extravasation conditions are generally more favourable. Specifically, cells with a stiffness of G = 270 μN/m have an extravasation window span of 9.75 μm at a location of 90.23 µm. In contrast, cells with a stiffness of G = 90 μN/m have a window span of 6.12 μm at a location of 92.6 µm. However, this wider window span for stiffer cells is accompanied by an increase in the minimum velocity by 17.73% and a decrease in the total adhesion force by 11.3%, which paradoxically may make extravasation more challenging despite the longer window of opportunity.

## 4 Discussion

Metastatic disease, a leading contributor to mortality from cancer, is closely associated with the dynamics of CTCs within the circulatory system. The blood flow hemodynamic conditions, and CTC properties, including the elasticity of the cell, along with the microvessel configurations are pivotal factors that regulate the transportation of CTCs and their potential to form secondary tumors. The architectural intricacies of the vasculature, including vessel diameter, curvature, branching patterns, and the presence of bifurcations, generate a diverse array of fluid dynamic environments. These conditions impose varying levels of shear stress and flow dynamics, which can critically affect the adhesion, survival, and migration of CTCs in the bloodstream.

Within the uniform environment of a straight vessel, blood flow facilitates relatively consistent and predictable interactions between CTCs and the vessel wall. Softer CTCs, which are slightly stiffer than RBCs, tend to follow the train of RBCs rather than adhere, suggesting a prolonged survival in the circulatory system (Lenarda et al., 2019). CTCs of intermediate softness exhibit a rolling behaviour, a dynamic facilitated by the interaction between P-selectin glycoprotein ligand-1 (PSGL-1) on the CTC surface and endothelial P-selectin, particularly in the company of surrounding RBCs. However, within narrower vessels where the diameter is less than 10 μm, the deformability of cancer cells enhances their ability to firmly adhere, potentially aiding in metastatic colonization (Dabagh et al., 2020). Conversely, the intricate nature of vascular networks, such as curvature and branching points introduces a range of hemodynamic complexities. We observed that the diversity in shear stress levels can subject CTCs to different mechanical stimuli, impacting their physical integrity and behavioural responses, such as cellular deformation, cell velocity, and the likelihood of adhesion or detachment from the endothelium.

Understanding the role of vessel configuration especially the curves and twists commonly found in smaller arteries, capillaries, and within tumors (Han, 2012), is of paramount importance as it may pave the way not only for unravelling the mechanisms of CTC dissemination but also for developing targeted therapeutic strategies to mitigate complications with the progression of metastasis. Our results suggest quantitatively that the presence of intricacies in the vessel configuration may contribute to metastasis progression, and we quantitively showed the underlying mechanisms responsible for the phenomenon. The variation in wall shear stress along curved vessels, as demonstrated in our computational model, provides valuable insights into the preferential and potential sites for CTC extravasation. This variation in the WSS suggests that alterations in flow dynamics due to curvature play a crucial role in the functioning of endothelial cells and may have implications for vascular health (Traub and Berk, 1998; Ko and McCulloch, 2001; Hur et al., 2012; Dabagh et al., 2014). Additionally, the level of vascular endothelial growth factor (VEGF) expression is closely linked to the WSS (Dela Paz et al., 2012). As WSS increases, there may be an upregulation of VEGF expression. This overexpression can facilitate the process of extravasation, where elevated VEGF levels lead to the expansion of gaps between neighbouring endothelial cells and the degradation of the endothelial surface layer (glycocalyx), enabling cells to penetrate through the vessel wall (Fan et al., 2011; Cai et al., 2012; Fan and Fu, 2016). The interplay between mechanical forces, such as the WSS and biochemical factors like VEGF, is thus a key area of research for understanding vascular remodelling and disease. This work demonstrated that regions of high shear stress, typically found at the peak of the inferior inner wall of curved vessels in our model, present an increased risk for CTC adhesion and subsequent extravasation due to elevated endothelial expression of adhesion molecules, which is a direct response to the mechanical stimuli.

Additionally, upon quantitative analysis of cell motion in curved *versus* straight vessels, our findings reveal that CTCs travelling in the straight vessel undergo uniform cellular deformation, which can be attributed to consistent shear stress and uniform VEGF levels along the vessel length. This results in more predictable forces affecting cell adhesion to the vessel wall, with diminished variation in the effective adhesion surface area. Consequently, comparing to curved vessels, the interaction dynamics between CTCs and endothelial cells in straight vessels are more predictable, simplifying modelling and understanding from a clinical perspective.

In contrast, the curvature of a curved vessel causes the flow to be asymmetrical, displacing the maximum velocity towards the inner inferior wall at the peak of microvessel. This skewness in the velocity profile aligns with the increased WSS observed in the same location, which may alter endothelial cell exposure to shear stress. Therefore, as CTCs move through regions of varying WSS, they encounter varying forces that cause notable deformations. This leads to differential mechanical stimuli that can affect cellular adhesion and signalling. Comparing to a straight vessel, the adhesion dynamics is not uniform along the vessel, with forces and effective adhesion areas varying along the vessel due to the localized shear stress distribution, increasing in high-shear regions and decreasing in low-shear regions. Therefore, in regions of high shear stress, where the CTC movement decelerates, there is an increase in bond formation, possibly due to the increased lifetime of previously formed bonds that allow the CTC to have more time to form interactions with the vessel wall. This increase in bond formation and adhesion force along with the reduction in the axial velocity of the CTC is in favour of extravasation, potentially raising the likelihood of tumor cells to form metastases in high-shear regions. The variation in the axial velocity of the CTC passing through the curved vessel is further inversely correlated with the number of molecular bonds formed between the cells and the vessel wall. This implies that in low-shear regions where cells travel faster, fewer bonds are formed. This is most likely a result of fewer endothelial receptors being engaged due to the reduced mechanical stress on the endothelial cells. This fluctuation in the total bond numbers when CTC passing through the curved vessel contrasts with the relatively constant number of molecular bonds seen in straight vessels, mirroring the uniform mechanical conditions. Furthermore, this variability in mechanical stimuli can have profound effects on the cellular behaviour, potentially influencing processes like intravascular migration, extravasation, and metastasis.

Furthermore, our analysis revealed significant differences in the behaviour of CTCs within straight and post-curvature straight sections of a vessel. Notably, CTCs were observed to traverse at a higher average axial velocity in the straight section following a curve in the vessel (region VII). This can be attributed to the reduction in the number of adhesion bonds at the end of the curvature due to low shear stress (region VI). Moreover, in curved vessels, the interactions of CTCs with the vessel wall and their impact on the temporal WSS distribution vary significantly. The proximity of cells to the wall in curved regions dynamically modulates WSS, affecting the local hemodynamic condition and potentially the biological responses of the endothelium. The coexistence of slowing down the cell motion and increased number of adhesions suggests that high-shear stress regions provide a promising environment for cell firm adhesion and possibly extravasation. Furthermore, the maximum WSS in the high-shear region is distributed over a larger surface area compared to regions with lower shear. This suggests that the slowed movement of the cells and the increased number of bonds allow for more substantial interaction with the blood flow, thereby amplifying the WSS. The straight vessel, on the other hand, exhibits a more uniform WSS distribution, hinting at a more consistent interaction between CTCs and the endothelium along the vessel.

In addition, the influence of vascular tortuosity on CTC dynamics is of utmost significance. Increased tortuosity, characterized by more pronounced vessel curvature, can amplify WSS in high-shear regions as well as diminish WSS in low-shear regions. Also, a higher tortuosity prolongs the transit time of cells through the vessel curvature due to increased pathways. Our results suggest that increased tortuosity can lead to greater cellular deformation and slower cellular movement in high-shear regions due to the increased adhesion bonds and total adhesion force, and potentially higher rates of extravasation. The amplified deformation of the cell and other changes in the adhesion behaviours are likely a result of the change in hydrodynamic forces and the adaptive response of the cell to the cumulative biomechanical stress within the tortuous vessel pathway. Therefore, our observation regarding the CTC mechanical response in various vascular tortuosity suggests that the tortuosity index may serve as a predictive measure and may provide a quantitative link between the vessel geometry and the biophysical behaviour of CTCs.

Our study also explored the modulation of the wall shear stress with the power parameter ( $P_{off}$ ) and its impact on endothelial receptor rupture, which may cause CTC detachment in low shear and firm adhesion in high shear regions due to a doubling in the number of adhesion bonds. In general, with an increase in the power parameter negativity, there are a discernible increase in bond rupture and acceleration in cellular movement within low-shear stress regions. Conversely, this change enhances bond stability and leads to a reduction in cellular velocity within regions of high shear stress. Furthermore, our investigation into the cell deformation has indicated that it is significantly influenced by the shear stress conditions. An increase in the negative value of $P_{off}$ correlates with a reduced cellular AR in low-shear regions, reflecting a reduction in cellular deformation. In contrast, high shear regions experience the opposite effect; an increase in $P_{off}$ results in a higher AR, suggesting an increased cellular deformation due to more persistent interactions with the vessel wall. These findings highlight the intricate balance between the mechanical forces exerted by the blood flow and cellular responses, where even subtle changes in the parameters governing these forces can drastically alter the fate of a CTC.

Furthermore, our investigation emphasizes on the role of cell stiffness, quantified by the shear modulus, on CTC’s ability to deform, adhere to surfaces, and detach under shear stress. The stretch tests conducted on CTCs with different levels of stiffness demonstrated a significant correlation between cell deformability and shear modulus. This, in turn, impacts many biomechanical responses of the cells, including aspect ratio, velocity, and adhesion forces. The softer CTCs exhibit significant deformability, which facilitates a larger surface area for interaction with the vessel wall, potentially leading to an increased adhesion force and chance of extravasation. However, this advantage can be compromised in low-shear regions where very soft CTCs are prone to detachment due to potentially increased bond rupture rates, which could lead to detachment, as detailed in the Results section. On the other hand, stiffer cells, show a reduced ability to deform compared to the softer cells while passing through the curved vessel. This altered behaviour contributes to reduction in the adhesion force and increase in the minimum velocity of the CTC, critical factors in reducing the likelihood of extravasation. Therefore, our finding indicates there exists an optimal stiffness level for successful extravasation, as cells that are too soft or too rigid are less likely to exit the vasculature successfully. This finding using our simplified model suggests that the view that softer cells always extravasate more efficiently may be an oversimplification and that there is a specific shear modulus range that maximizes the metastatic potential of CTCs in curved vessels.

Our model has a limitation due to absence of RBCs in the domain. Lenarda et al. (2019) suggested that malignant tumor cells with stiffness less than or equal to red blood cells (RBCs) are not able to marginate. Therefore, in situations involving RBCs, CTCs which are stiffer than RBCs would marginate toward the vessel walls. Within this particular situation, our observation of the detachment possibility of most deformable cells, which are still stiffer than RBCs, could be lowered by the presence of RBCs, which could keep them close to the vessel wall rather than allowing them to flow freely in the blood plasma. Future studies should integrate RBCs in the model to study this phenomenon more thoroughly. Moreover, future incorporation of RBCs into the model can also facilitate the application of non-Newtonian behaviour of blood, thereby enhancing the realism of the simulation.

Furthermore, we did not consider several factors that are known to critically influence cell adhesion. Specifically, the association rate of adhesion molecules, the presence platelets in blood plasma, and the bond elasticity were not included in our model. Additionally, the impact of the nucleus within CTCs and the cytoplasmic viscosity, both of which are integral to cellular behavior, were not addressed. Complexities within the domain geometries, such as bifurcations, and the effects of varying vessel diameters on adhesion were also beyond the scope of this study. Moreover, in this study only one CTC size (diameter of 8 µm) was investigated. To provide a comprehensive understanding of the adhesion behaviour within the microcirculatory environment, the impact of these factors should be further investigated in future studies.

In conclusion, our computational model provides a framework to analyze the underlying mechanisms for CTC traversing through microcirculation, alongside the adhesion dynamics of CTC to endothelial cells in curved microvessels. Notably, the main findings of this study in terms of the values of variables, are summarized in Table 2. Therefore, this model enables us to achieve a comprehensive understanding of the mechanical characteristics of CTCs in physiological conditions, where the mechanical properties of vessels can influence the ability of CTCs to adhere, survive, and eventually extravasate.

**TABLE 2:** Quantified summary of findings.

Findings	Description
**Role of Vessel configuration (curved vessel vs straight vessel)**	**WSS variation:**
	• Decrease by approximately 25% in low-shear regions (Regions II and VI)
	• Increase by approximately 58.5% in high-shear region (Region IV)
	**Adhesion Force on CTC**
	• Decrease in low-shear regions (61.52 ± 5.22 pN in Region II and 63.79 ± 5.71 pN in region VI) compared to the straight vessel (92.52 ± 2.8534 pN)
	• Increase in the high-shear region (161.36 ± 8.76 pN in Region IV)
	**Median Number of Adhesion Bonds**
	• Lowest in low-shear regions (∼5–6) compared to the straight vessel (∼12)
	• Highest in high-shear region (∼40)
	**Taylor’s Aspect Ratio**
	**•** Decrease in low-shear regions (∼0.07) of the curved vessel compared to the straight vessel (∼0.075)
	**•** Increase in high-shear region (∼0.11)
	**Axial Velocity of CTC**
	• Highest in low-shear regions (∼0.84 mm/s)
	• Lowest in high-shear regions (∼0.29 mm/s)
	• CTCs traverse faster with an average axial velocity of 0.8 mm/s in post-curvature straight sections (Region VII) compared to 0.68 mm/s in entirely straight vessels
**Wall Shear Stress Modulation with Power Parameter (** $P_{off}$ )	**Modulation of bond rupture rate** by doubling $P_{off}$ value
	• Up to 33.3% increase in the low-shear region
	• Up to 20% decrease in high-shear region
	**Mode number of adhesion bonds** by decreasing $P_{off}$ from −3 to −5
	• Decrease from 5 to 4 in the low-shear regions
	• Increase from 43 to 70 bonds in the high-shear region
	**Taylor’s Aspect Ratio** by decreasing $P_{off}$ from −3 to −5
	• Decrease from 0.068 to 0.063 in low-shear regions
	• Increase from 0.116 to 0.125 in high-shear regions
	**Axial cell velocity** by decreasing $P_{off}$ from −3 to −5
	• Increase from 0.792 mm/s to 0.842 mm/s in low-shear regions
	• Decrease from 0.3118 mm/s to 0.12618 mm/s in high-shear regions
	⁃ Further increases in $P_{off}$ to −10 and −20 cause significant cell detachment and acceleration in low-shear regions, and the subsequent establishment of new bonds in high-shear regions leads to firm adhesion
**Effect of Tortuosity Index (TI)**	**WSS variation** by doubling domain amplitudes from *a = D* to *a = 2D*
	• 50% increase in maximum WSS ratio and 15.3% decrease in minimum WSS ratio
	**Transit time** by increasing TI from 1.06 to 1.68
	• Prolonged transit time through vessel curvature (from 0.026 to 0.0411 ms)
	**Extravasation Window Span** by increasing TI from 1.06 to 1.68
	• Shift closer to the peak of the curved vessel, from approximately *x =* 94 µm within the range of ∼90–100 µm to around ∼89.5 µm, within a narrower range of ∼87–90 µm
	**Adhesion Force** by increasing TI from 1.06 to 1.68
	• Decrease from 94.5 pN to 68.88 pN at low-shear region
	• Increase from 151 pN to 409 pN at the high-shear region
	**Taylor’s Aspect Ratio** by increasing TI from 1.06 to 1.68
	• Reduce in the low-shear region from 0.08 to 0.068
	• Increase in the high-shear region from 0.12 to 0.1276
	• Change in the aspect ratio (ΔAR) in the curved vessel from 0.5 to 0.62
	**Minimum Cell Velocity** in curved vessel by increasing TI from 1.06 to 1.68
	• Decrease from 0.32 mm/s to 0.0809 mm/s
**Effect of CTC stiffness**	**Axial velocity** by increasing *G* from 90 μN/m to 270 μN/m
	• Decrease from 0.77 mm/s to 0.71 mm/s in low-shear regions
	• Increase from 0.27 mm/s to 0.32 mm/s in high-shear region
	**Adhesion Force** by increasing *G* from 90 μN/m to 270 μN/m
	• Increase from 46 pN to 60 pN in low-shear regions
	• Decrease from 216 pN to 191 pN in high-shear region
	**Taylor’s Aspect Ratio** by increasing *G* from 90 μN/m to 270 μN/m
	• Decrease in the maximum aspect ratio from 0.12 to 0.066 in the high-shear region
	• Decrease in *ΔAR* from 0.53 to 0.4
	**Extravasation Window Span** by increasing *G* from 90 μN/m to 270 μN/m
	• Shift closer to the peak of the curved vessel, from *x =* 92.6 µm within the span of 6.12 µm to *x =* 90.23 µm with a wider span of 9.75 µm
	⁃ Very soft CTCs (*G =* 30 μN/m) are prone to detachment due to potentially increased bond rupture rates. However, this may be influenced by the limitations of our model due to the absence of RBCs in the simulated domain

## Data Availability

The raw data supporting the conclusion of this article will be made available by the authors, without undue reservation.
