# Supplementary figures and images for: Computational analysis of cancer cell adhesion in curved vessels affected by wall shear stress for prediction of metastatic spreading
Source: Front Bioeng Biotechnol. 2024 May 27;12:1393413. doi: 10.3389/fbioe.2024.1393413 (PMC11163055; doi:10.3389/fbioe.2024.1393413)

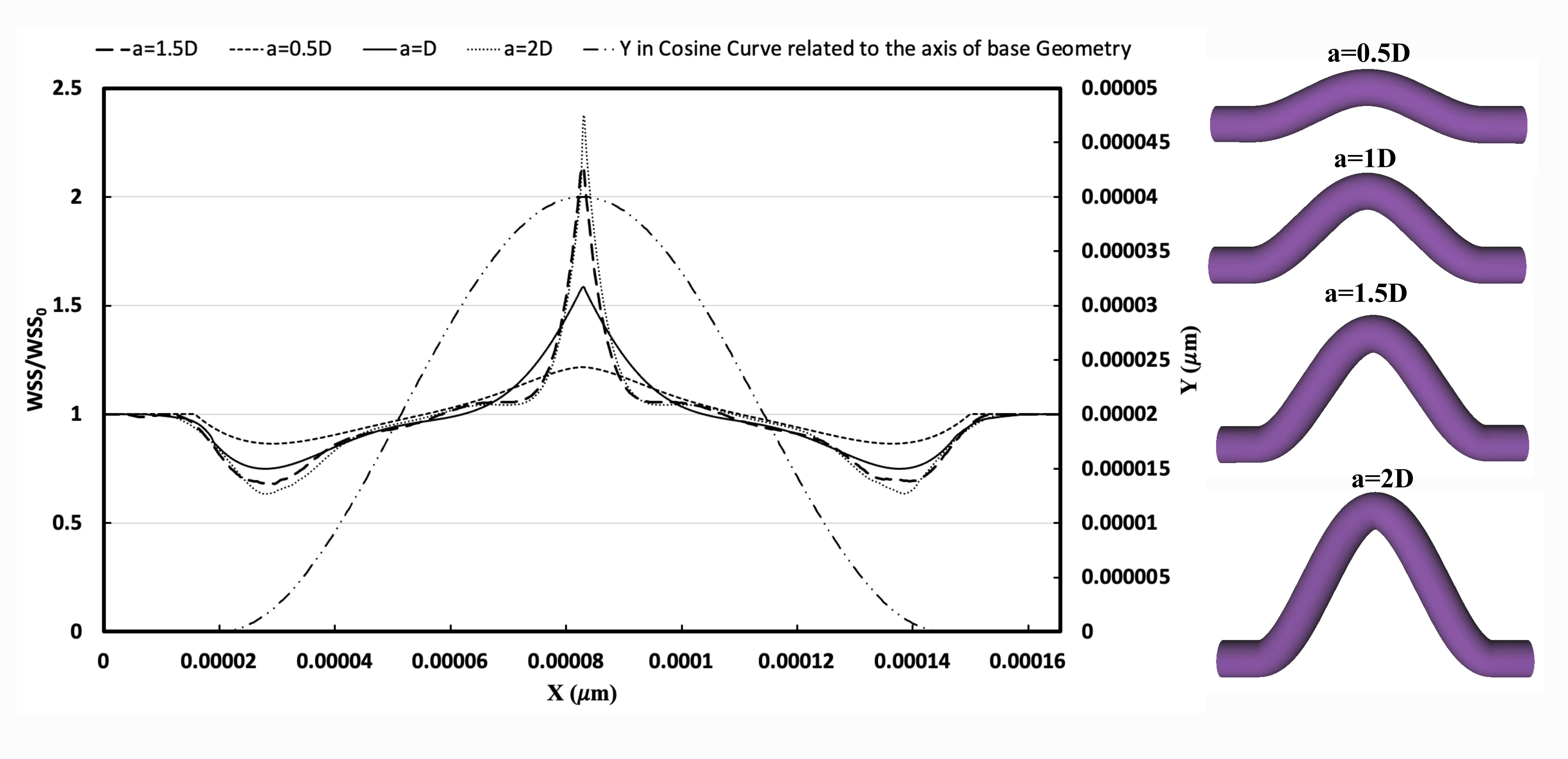

Supplement: Supplementary file 1 [file Image3.TIFF]

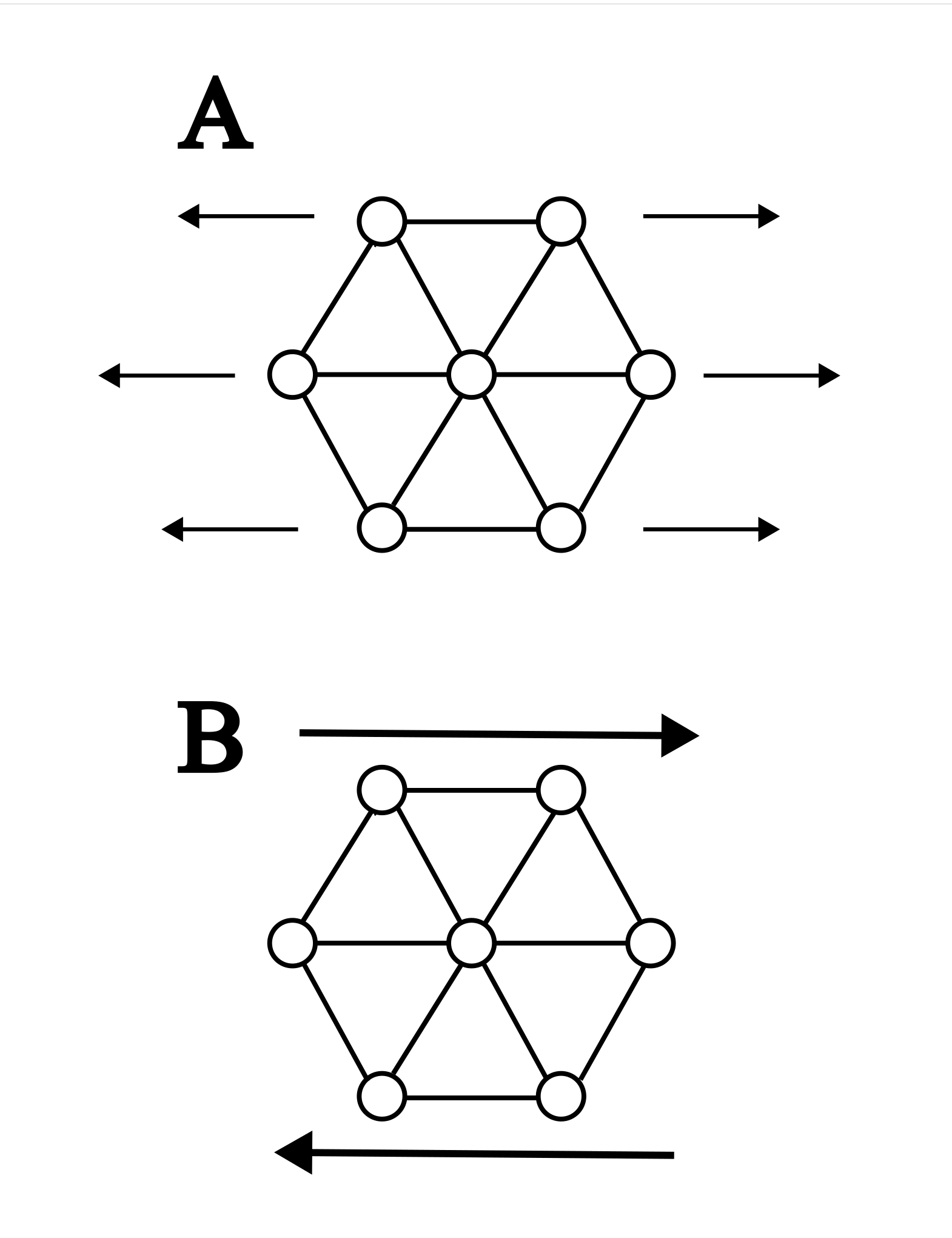

Supplement: Supplementary file 2 [file Image1.tiff]
