# Supplementary material for: Computational analysis of cancer cell adhesion in curved vessels affected by wall shear stress for prediction of metastatic spreading
Source: Front Bioeng Biotechnol. 2024 May 27;12:1393413. doi: 10.3389/fbioe.2024.1393413 (PMC11163055; doi:10.3389/fbioe.2024.1393413)
Supplement: Supplementary file 4 [file DataSheet1.docx]

Supplementary Material

# Supplementary Figures


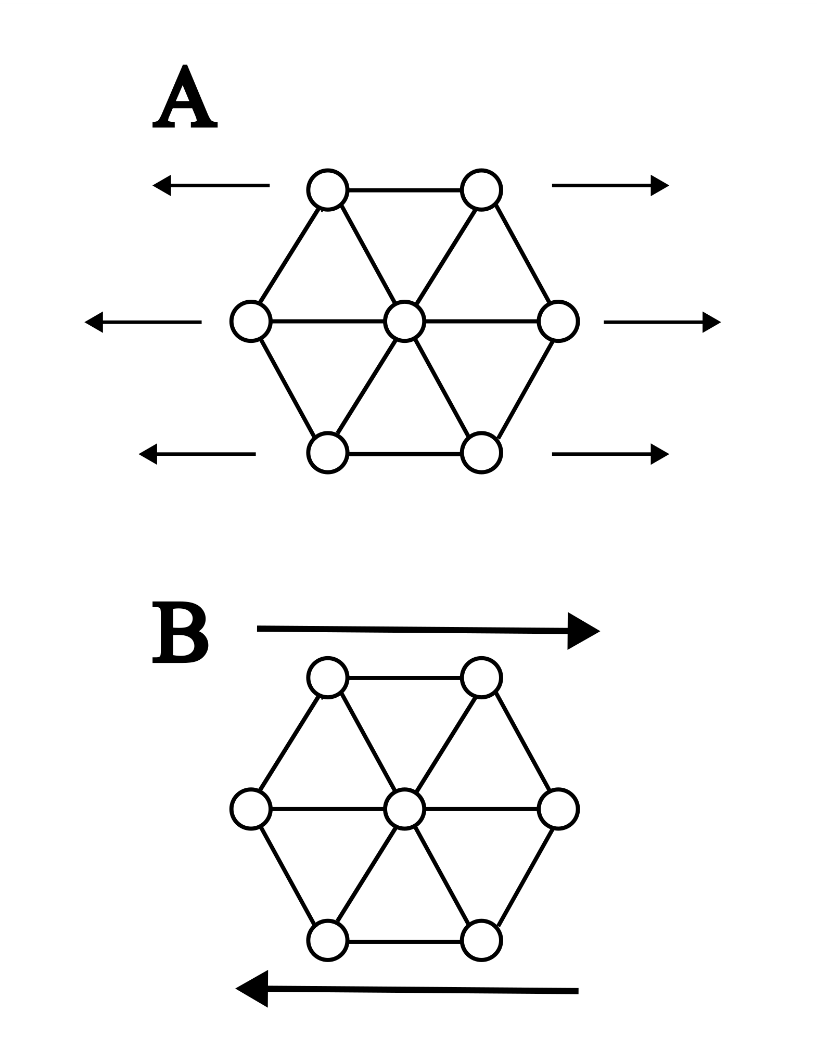


**Supplementary Figure 1.** Mechanical tests on a hexagonal patch of membrane. **(A)** uniaxial stretching. **(B)** shearing, after Závodszky et al. (2017) under [CC-BY 4.0](https://creativecommons.org/licenses/by/4.0/) license.


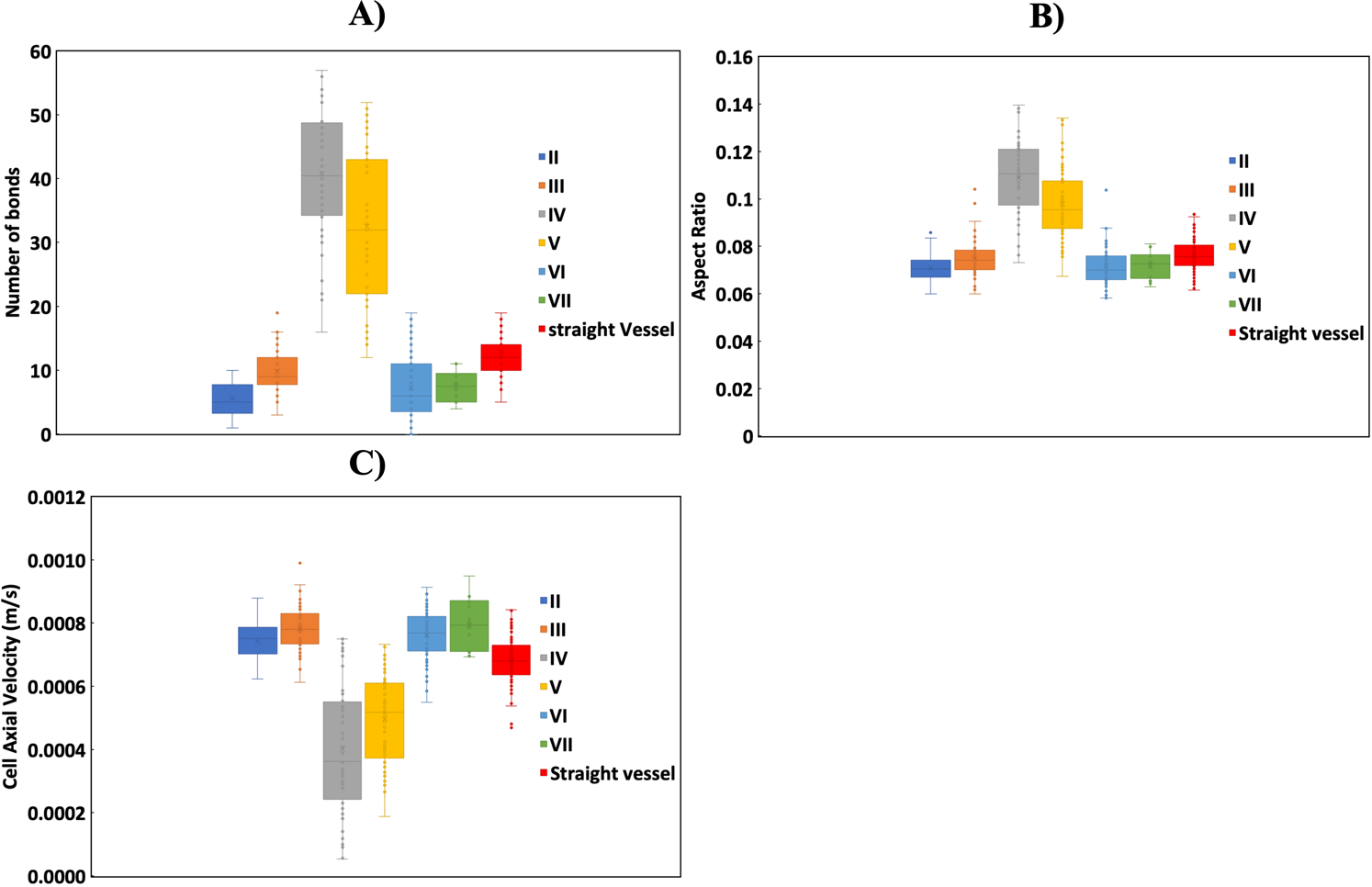


**Supplementary Figure 2.** Comparison of the behaviour of CTCs in the straight vessel with that in the regions of the curved vessel in terms of **(A)** the number of adhesion bonds, (**B)** aspect ratio, and (**C)** cell axial velocity.


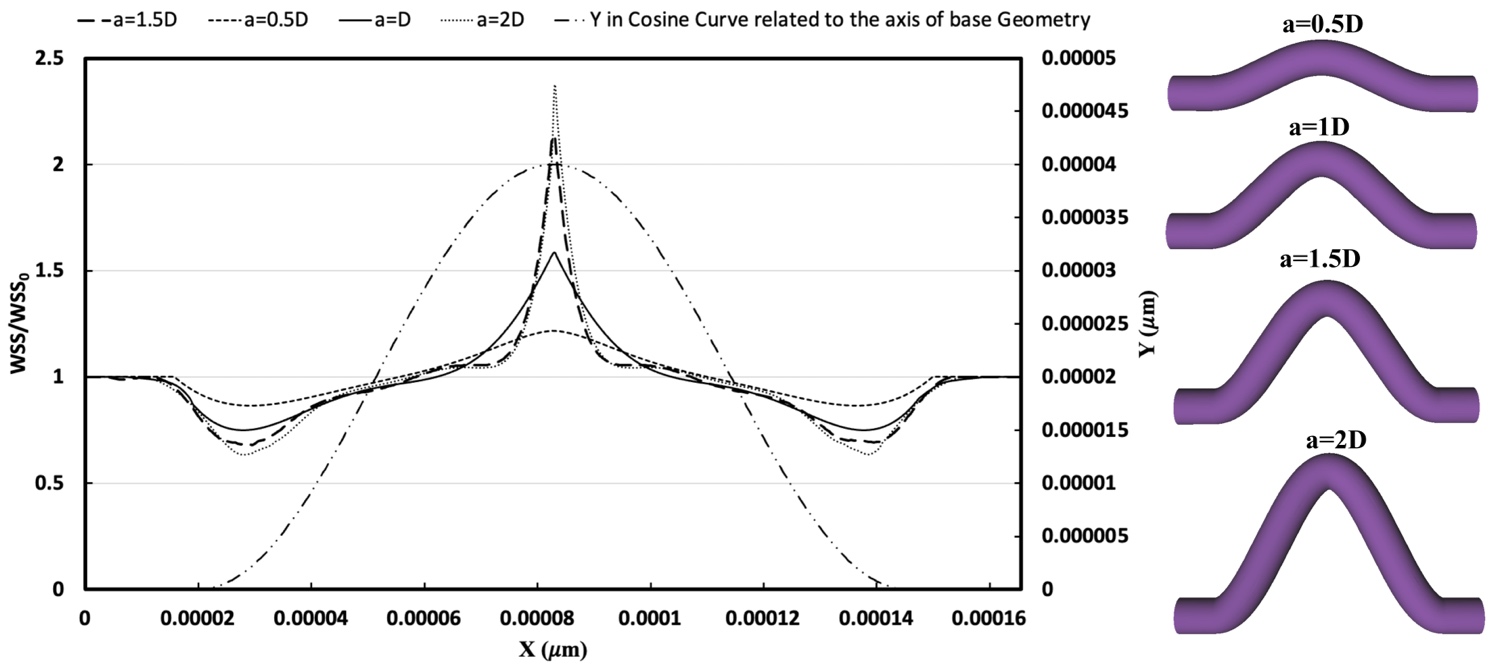


**Supplementary Figure 3.** Spatial distribution WSS ratio on the inferior curvature in the curved vessel with various amplitude magnitudes (a) relative to a straight one considering pure blood flow without any cell with the same Reynolds number. Various geometries of the curved vessel related to different amplitudes are depicted on the right.

# Supplementary Table

Supplementary Table 1 **Parameters used in the model.**

| **Parameter** | **Definition** | **Physical value** | **References** |
| --- | --- | --- | --- |
| $T$ | Absolute temperature | $310 K$ | (Chang and Hammer, 1996) |
| $k_{f}^{0}$ | Unstressed forward reaction rate | $750 s^{-1}$ | (Zhang et al., 2018) |
| P_off_ | Power parameter for modulating the effect of shear stress on the rate of bond rupture | -3 | (Yan et al., 2012) |
| P_on_ | Power parameter for modulating the effect of shear stress on the rate of bond formation | 1 | (Yan et al., 2012) |
| $k_{r}^{0}$ | Unstressed reverse reaction rate | $20 s^{-1}$ | (Zhang et al., 2018) |
| $k_{l_{\mathrm{CTC}}}$ | Link force coefficient (equivalent to G=90μN/m) | 150 | (Lenarda et al., 2019) |
| $k_{b_{\mathrm{CTC}}}$ | Bending force modulus | 600 $K_{B}T$ | (Cui et al., 2021) |
| $k_{a_{CTC}}$ | Local area conservation coefficient (equivalent to Poisson's Ratio of 1/3) | 250 | (Tan, 2015) |
| $k_{v_{\mathrm{CTC}}}$ | Volume conservation coefficient^1^ | 600 | - |
| $\sigma_{ts}$ | Transition state spring constant | ${2\times10}^{-6}\frac{N}{m}$ | (Zhang et al., 2018) |
| $\sigma_{b}$ | Bond spring constant | ${10}^{-4}\frac{N}{m}$ | (Zhang et al., 2018) |
| $l_{0}$ | Equilibrium bond length in adhesive dynamics model | $0.5 \mu m$ | (Dabagh et al., 2020) |
| $H_{c}$ | Cut-off length for bond formation | $1 \mu m$ | (Dabagh et al., 2020) |
| $K_{B}$ | Boltzmann constant | $1.38\times{10}^{-23} J/K$ | (Dabagh et al., 2020) |
| $\Delta t$ | Time interval | ${10}^{-7} s$ | (Wang et al., 2013; Wu and Qi, 2019) |
| $\dot{\gamma}$ | Fluid Average Shear rate (${U_{max}}/D$) | 200 | (Zhang et al., 2018; Dabagh et al., 2020) |
| $\Delta x$ | Fluid lattice resolution | $0.5 \mu m$ | (Lenarda et al., 2019) |
| $D_{cp}$ | Microvessel diameter | $20 \mu m$ | (Lenarda et al., 2019) |
| $L_{cp}$ | Length of the periodic microvessel domain | $165 \mu m$ | - |
| $\nu$ | Kinematic viscosity of plasma | $1.2\times{10}^{-6} {m^{2}}/s$ | (Dabagh and Randles 2019) |
| $\mu$ | Dynamic viscosity of plasma | $1.2 mPa.s$ | (Dabagh and Randles 2019) |
| $r_{CTC}$ | CTC baseline radius | $4 \mu m$ | (Suresh 2007; Anvari et al.2021) |

1. **^Volume modulus is chosen to be large but numerically stable (Závodszky et al., 2017).^**

# References

Anvari, S., Osei, E., and Maftoon, N. (n.d.). Interactions of Platelets with Circulating Tumor Cells Contribute to Cancer Metastasis. *Sci Rep*.

Chang, K.-C., and Hammer, D. A. (1996). Influence of Direction and Type of Applied Force on the Detachment of Macromolecularly-Bound Particles from Surfaces. *Langmuir* 12, 2271–2282. doi: 10.1021/la950690y

Cui, J., Liu, Y., Xiao, L., Chen, S., and Fu, B. M. (2021). Numerical study on the adhesion of a circulating tumor cell in a curved microvessel. *Biomech Model Mechanobiol* 20, 243–254. doi: 10.1007/s10237-020-01380-x

Dabagh, M., Gounley, J., and Randles, A. (2020). Localization of Rolling and Firm-Adhesive Interactions Between Circulating Tumor Cells and the Microvasculature Wall. *Cel. Mol. Bioeng.* 13, 141–154. doi: 10.1007/s12195-020-00610-7

Dabagh, M., and Randles, A. (2019). Role of deformable cancer cells on wall shear stress-associated-VEGF secretion by endothelium in microvasculature. *PLOS ONE* 14, e0211418. doi: 10.1371/journal.pone.0211418

Késmárky, G., Kenyeres, P., Rábai, M., and Tóth, K. (2008). Plasma viscosity: A forgotten variable. *Clinical Hemorheology and Microcirculation* 39, 243–246. doi: 10.3233/CH-2008-1088

Lenarda, P., Coclite, A., and Decuzzi, P. (2019). Unraveling the Vascular Fate of Deformable Circulating Tumor Cells Via a Hierarchical Computational Model. *Cel. Mol. Bioeng.* 12, 543–558. doi: 10.1007/s12195-019-00587-y

Suresh, S. (2007). Biomechanics and biophysics of cancer cells. *Acta Biomaterialia* 3, 413–438. doi: 10.1016/j.actbio.2007.04.002

Tan, J. (2015). Lattice Boltzmann Method and Its Applications in Soft Matter. 166.

Wang, W., Mody, N. A., and King, M. R. (2013). Multiscale model of platelet translocation and collision. *Journal of Computational Physics* 244, 223–235. doi: 10.1016/j.jcp.2012.08.014

Wu, T.-H., and Qi, D. (2019). Investigation of shear rates of rolling adhesion on leukocytes with bending of microvilli. *Phys. Rev. Fluids* 4, 063101. doi: 10.1103/PhysRevFluids.4.063101

Yan, W. W., Cai, B., Liu, Y., and Fu, B. M. (2012). Effects of wall shear stress and its gradient on tumor cell adhesion in curved microvessels. *Biomech Model Mechanobiol* 11, 641–653. doi: 10.1007/s10237-011-0339-6

Závodszky, G., van Rooij, B., Azizi, V., and Hoekstra, A. (2017). Cellular Level In-silico Modeling of Blood Rheology with An Improved Material Model for Red Blood Cells. *Front. Physiol.* 8, 563. doi: 10.3389/fphys.2017.00563

Zhang, Z., Du, J., Wei, Z., Wang, Z., and Li, M. (2018). Effects of membrane deformability and bond formation/dissociation rates on adhesion dynamics of a spherical capsule in shear flow. *Biomech Model Mechanobiol* 17, 223–234. doi: 10.1007/s10237-017-0956-9
